# Foreskin cutting beliefs and practices and the acceptability of male circumcision for HIV prevention in Papua New Guinea

**DOI:** 10.1186/1471-2458-13-818

**Published:** 2013-09-09

**Authors:** David MacLaren, Rachael Tommbe, Tracie Mafile’o, Clement Manineng, Federica Fregonese, Michelle Redman-MacLaren, Michael Wood, Kelwyn Browne, Reinhold Muller, John Kaldor, William John McBride

**Affiliations:** 1 School of Medicine and Dentistry, James Cook University, McGregor Road, Smithfield, Cairns 4878, Queensland, Australia; 2 School of Health Science, Pacific Adventist University, Port Moresby, National Capital District, Papua New Guinea; 3 Deputy Vice Chancellor, Pacific Adventist University, Port Moresby, National Capital District, Papua New Guinea; 4 Faculty of Health Science, Divine Word University, Madang, Madang Province, Papua New Guinea; 5 Global Health Unit, University of Montreal Hospital Research Centre, Montreal, Quebec, Canada; 6 School of Arts and Social Science, James Cook University, Cairns, Queensland, Australia; 7 Rural Primary Health Services Delivery Project, National Department of Health, Port Moresby, Papua New Guinea; 8 School of Public Health, Tropical Medicine and Rehabilitation Science, James Cook University, Cairns, Queensland, Australia; 9 Tropical Health Solutions, Townsville, Australia; 10 Kirby Institute, University of New South Wales, Sydney, New South Wales, Australia

**Keywords:** Male circumcision, Acceptability, Foreskin cutting, Papua New Guinea, HIV, Prevention, Beliefs, Foreskin cutting practices, Longitudinal Foreskin cut, Circumferential Foreskin cut

## Abstract

**Background:**

Male circumcision (MC) reduces HIV acquisition and is a key public health intervention in settings with high HIV prevalence, heterosexual transmission and low MC rates. In Papua New Guinea (PNG), where HIV prevalence is 0.8%, there is no medical MC program for HIV prevention. There are however many different foreskin cutting practices across the country’s 800 language groups. The major form exposes the glans but does not remove the foreskin. This study aimed to describe and quantify foreskin cutting styles, practices and beliefs. It also aimed to assess the acceptability of MC for HIV prevention in PNG.

**Methods:**

Cross-sectional multicentre study, at two university campuses (Madang Province and National Capital District) and at two ‘rural development’ sites (mining site Enga Province; palm-oil plantation in Oro Province). Structured questionnaires were completed by participants originating from all regions of PNG who were resident at each site for study or work.

**Results:**

Questionnaires were completed by 861 men and 519 women. Of men, 47% reported a longitudinal foreskin cut (cut through the dorsal surface to expose the glans but foreskin not removed); 43% reported no foreskin cut; and 10% a circumferential foreskin cut (complete removal). Frequency and type of cut varied significantly by region of origin (p < .001). Most men (72-82%) were cut between the ages of 10 – 20 years. Longitudinal cuts were most often done in a village by a friend, with circumferential cuts most often done in a clinic by a health professional. Most uncut men (71%) and longitudinal cut men (84%) stated they would remove their foreskin if it reduced the risk of HIV infection. More than 95% of uncut men and 97% of longitudinal cut men would prefer the procedure in a clinic or hospital. Most men (90%) and women (74%) stated they would remove the foreskin of their son if it reduced the risk of HIV infection.

**Conclusion:**

Although 57% of men reported some form of foreskin cut only 10% reported the complete removal of the foreskin, the procedure on which international HIV prevention strategies are based. The acceptability of MC (complete foreskin removal) is high among men (for themselves and their sons) and women (for their sons). Potential MC services need to be responsive to the diversity of beliefs and practices and consider health system constraints. A concerted research effort to investigate the potential protective effects of longitudinal cuts for HIV acquisition is essential given the scale of longitudinal cuts in PNG.

## Background

Male circumcision (MC), or the surgical removal of the foreskin, has received intense public health attention since three large randomised trials, published between 2005 and 2007, reported that the procedure can reduce male susceptibility to heterosexual acquisition of HIV by approximately 60% [1-3]. These results confirmed earlier observational studies that documented an association between lower HIV infection rates and MC [4,5]. In 2007 it was estimated that up to 5.7 million new HIV infections could be averted over 20 years through the implementation of MC services in sub-Saharan Africa [6-8]. WHO and UNAIDS now recommend MC programs be included in comprehensive HIV prevention packages in settings of high HIV prevalence with heterosexual transmission and low MC rates [9].

MC for HIV prevention is viewed favourably across numerous high HIV prevalence settings in East and Southern Africa, both in traditionally circumcising and non-circumcising communities [10-21]. MC programs are now being implemented across this region, accompanied by ongoing acceptability studies [12,22-35]. However, a detailed understanding of local social, cultural, gender, religious and medical issues must underpin such MC programs [36-39].

MC may also play an important public health role in moderate HIV prevalence countries. However very few investigations of MC’s acceptability, feasibility and epidemiological impact have been conducted in such settings [40-46]. Papua New Guinea (PNG), the largest South Pacific Island country, has the second highest HIV prevalence of the Asia-Pacific region (after Thailand). In 2012 PNG had an estimated adult HIV seroprevalence of 0.79% (15–49 years), associated with widespread behavioural risk and high rates of sexually transmitted infections [47-49]. More females than males are infected with HIV, suggesting heterosexual intercourse as the primary driver. The PNG health system does not provide routine MC services. Investigating MC for HIV prevention is a current research priority for the PNG National AIDS Council [50].

PNG has extreme social, cultural and geographical diversity. PNG’s 7.1 million people speak over 800 distinct languages and live in different settings: from villages on remote coral atolls to highland valleys to regional and provincial towns and their associated peri-urban squatter settlements. Reflecting this diversity, HIV prevalence is unevenly distributed: Highlands and Southern Regions have 0.89% and 0.88% respectively and Momase and New Guinea Islands Regions 0.66% and 0.58% respectively [47]. PNG’s unparalleled diversity means national HIV policy formulation is complex and requires evidence from a wide range of studies [51,52].

The context of MC in PNG is also complex. Most cultural groups do not traditionally practice MC, however there is a wide variety of foreskin cutting practices across the country [53,54]. The full removal of the foreskin, commonly referred to as a ‘round cut’, is produced by a circumferential cut to the foreskin and produces results equivalent to medical MC. Local variations, with many local descriptive names, are produced by a longitudinal cut along the dorsal surface of the foreskin. This exposes the glans penis and leaves the foreskin hanging loose beneath the penis. These variations are often generically referred to as ‘straight cut’ or ‘split’. Most of these cuts take place in the community with few occurring through the formal health system [55-57]. The rich ethnographic and anthropological record in PNG has detailed descriptions of initiation and blood-letting rituals in some cultural groups that involve multiple cuts to the foreskin and penis [58-60].

In recent years there appears to have been a shift in foreskin cutting practices in PNG that have paralleled dramatic social, economic and religious change [54,60]. A National HIV/AIDS Behavioural Surveillance Study in 2006 documented 26 – 70% of men had some form of foreskin cut, however the study did not differentiate between the ‘round cut’ or ‘straight cut’ [61]. More recent studies have made the distinction [62,63] and found 25 - 50% of men had some form of foreskin cut, with considerable diversity in the extent and type of foreskin cut reported. Qualitative studies are expanding knowledge about beliefs and practices of the various styles of foreskin cutting, and their implications for HIV prevention in PNG [45,46,57]. Men in these studies were generally in favour of MC being introduced for HIV prevention, but women were cautious or not in favour because of cultural and religious concerns and fear of sexual disinhibition of husbands or partners [45,46]. Building on these studies, it was important to quantify the diversity of foreskin cutting styles, practices and beliefs in PNG and assess the proportion of men and women who may find MC acceptable for HIV prevention.

This paper reports on the collaborative ‘Acceptability of Male Circumcision for HIV Prevention in PNG’ study carried out from July 2010 to February 2011. Here we report quantitative results for two objectives of the study: (i) describe and categorise male genital cutting, which includes MC; to ii) examine social, cultural and religious practices and their influence on the acceptability of MC for HIV prevention.

## Methods

This was an observational cross-sectional study, conducted in collaboration between researchers from Papua New Guinean and Australian universities and partnering with companies at two ‘rural development’ sites.

### Participants

The study was undertaken at four sites in four provinces: (i) Pacific Adventist University (PAU), Port Moresby, National Capital District; (ii) Divine Word University (DWU), Madang, Madang Province; (iii) Higaturu Oil Palms, Popondetta, Oro Province; and (iv) Porgera Joint Venture, Porgera, Enga Province. The first two sites are major universities that have predominantly residential student bodies. The latter two are a major oil palm production facility on the coastal plains and a major gold mine in the highlands. Sites were chosen to provide access to a wide a variety of socio-cultural, geographic, religious and educational backgrounds. All four sites attract people from across all PNG regions and most cultural backgrounds, to study or work. Key collaborators at each site had also been involved with previous HIV research or prevention programs.

A sample of 200 men per site was necessary for a precision of at least 5% to estimate the prevalence of male circumcision in a range of 5 – 50%. Further, a sample of 100 women per site was deemed feasible to collect data on women’s social, cultural and religious perspectives of foreskin cutting and the acceptability of MC for HIV prevention. Therefore, considering possible 20% attrition, at each site 250 males and 175 female were invited to enrol. At the two university campuses students were selected via a systematic sampling approach (by alphabetised student lists). Blank envelopes containing self-administered questionnaires were given to the selected students via regional student group leaders. At the two rural development sites men and women who attended the health centre for routine workplace health and safety checks or minor health issues were invited to participate sequentially, until the targeted sample size was reached and allowing for recruitment gaps if researchers were still engaged in assisting previous participants with the questionnaire. To achieve the sample size in women, recruitment also took place by inviting all women employed in selected company departments to participate.

### Questionnaires

The structured questionnaire contained eight sections and covered demographics, province of origin, knowledge and attitudes, sexual history and foreskin cutting/penile modification. Both closed and open ended questions were used. Questionnaires were offered in English or *Tok Pisin* (PNG lingua franca) for use in rural sites. The questionnaire had an information sheet attached that clearly stated that participation was voluntary and to complete and return the questionnaire meant consent was given for results to be used in the study. The male questionnaire contained a seven level classification of foreskin cutting. A photograph accompanied the written description for each of the seven foreskin cutting types with the statement ‘please circle the number beside the picture that looks most like your own foreskin’. See Additional file 1: Figure S1 for male questionnaire in English and Additional file 2: Figure S2 for female questionnaire in English. Questionnaires were generally self-administered but assistance was rendered at rural sites by a researcher of the same sex if literacy skills did not allow for self-administration. In addition to questionnaires, individual interviews and focus groups discussions were conducted. Findings from these qualitative methods will be published separately.

### Data handling and analysis

Collected questionnaires were collated and data were entered into an Excel spreadsheet which was subsequently imported into the statistical package SPSS (Version 20) for analysis. The actual data analysis was preceded by extensive plausibility checks and data cleaning procedures. Numerical information was summarized as percentages or mean and standard deviation or median and inter-quartile range. Bivariate analyses were undertaken by employing exact versions of standard test procedures such as exact binomial test of two categorical variables. Non parametric Kruskal-Wallis tests were used for comparison of numerical values between behavioural categories since the underlying distributions proved to be skewed.

### Ethics

Ethics clearance was granted by Human Research Ethics Committees of Pacific Adventist University, Divine Word University, James Cook University (Australia) and Papua New Guinea National AIDS Council. Endorsement was also provided by the Provincial AIDS Committees of the National Capital District and Oro, Enga and Madang Provinces.

Results were provided to institutions, key stakeholders and participants during interactive workshops at the four study sites between Oct 2011 and March 2012.

## Results

### Demographic characteristics of study population

The structured questionnaire was completed by a total of 1,380 participants (861 men and 519 women) at the four sites. DWU contributed 24% (n = 208) of men to the sample; PAU 24% (n = 204); Porgera 26% (n = 227) and Popondetta 26% (n = 222). For women, the respective proportions were DWU 20% (n = 103); PAU 30% (n = 157); Porgera 30% (n = 158) and Popondetta 20% (n = 101). Overall, age in men ranged from 18–65 years (median 25 IQR 21–32) and in women from 18–58 (median 24 IQR 21–30). The majority of participants at PAU and DWU were under 25 years of age (77% and 77% of men; 91% and 80% of women respectively). The majority of participants at Porgera and Popondetta were 25 year or older (88% and 63% of men; 82% and 75% of women respectively). Region of origin in the overall sample was distributed as follows: Highlands (men 47%; women 42%); Southern (men 27%; women 28%); Momase (men 18%; women 14%); New Guinea Islands (men 8%; women 16%) and reflects regional distribution from 2011 national population census (Highlands 43%; Momase 25%; Southern 19%; New Guinea Islands 14%) [64]. More details on socio-demographic information by Region of Origin are presented in Table 1.

**Table 1:** Demographic characteristics by region of origin

**Characteristic^**	**New Guinea Islands**		**Momase**		**Southern**		**Highlands**	
	**% (n)**		**% (n)**		**% (n)**		**% (n)**	
	**Male**	**Female**	**Male**	**Female**	**Male**	**Female**	**Male**	**Female**
	**n** **= 72**	**n = 80**	**n = 153**	**n = 72**	**n = 230**	**n = 146**	**n = 402**	**n = 218**
**Age**								
Under 25	50.8 (32)	66.2 (51)	61.9 (91)	70.6 (48)	48.1 (104)	48.9 (68)	45.9 (168)	47.4 (91)
25 and over	49.2 (31)	33.8 (26)	38.1 (56)	29.4 (20)	51.9 (112)	51.1 (71)	54.1 (198)	52.6 (101)
**Site**								
DWU	31.9 (23)	27.5 (22)	35.9 (55)	38.9 (28)	12.2 (28)	17.8 (26)	25.1 (101)	12.4 (27)
PAU	45.8 (33)	56.3 (45)	25.5 (39)	33.3 (24)	18.7 (43)	19.9 (29)	21.6 (87)	26.6 (58)
Porgera	16.7 (12)	8.8 (7)	10.5 (16)	12.5 (9)	5.2 (12)	6.2 (9)	46.5 (187)	60.6 (132)
Popondetta	5.6 (4)	7.5 (6)	28.1 (43)	15.3 (11)	63.9 (147)	56.2 (82)	6.7 (27)	0.5 (1)
**Marital status**								
Single	70.8 (51)	72.5 (58)	71.9 (110)	81.9 (59)	56.5 (130)	44.8 (65)	55.7 (224)	45.9 (100)
Married	27.8 (20)	21.3 (17)	26.8 (41)	15.3 (11)	40.9 (94)	49.7 (72)	42.3 (170)	44 (96)
Separated/Divorced	1.4 (1)	6.3 (5)	1.4 (2)	2.8 (2)	2.6 (6)	5.6 (8)	1.9 (8)	10.1 (22)
**Religion**								
Anglican	1.4 (1)	0 (0)	6.3 (6)	1.4 (1)	36.1 (83)	27.4 (40)	0.5 (2)	0 (0)
Catholic	31.9 (23)	22.5 (18)	21.1 (32)	18.3 (13)	6.1 (14)	9.6 (14)	14.4 (58)	7.4 (16)
Lutheran	0 (0)	0 (0)	28.3 (43)	18.3 (13)	1.7 (4)	3.4 (5)	13.7 (55)	4.6 (10)
Pentecostal	8.3 (6)	10 (8)	24 (15.8)	16 (22.5)	27.4 (63)	23.3 (34)	24.1 (97)	33.2 (72)
Seventh day Adventist	45.8 (33)	55 (44)	23.6 (40)	33.8 (24)	16.5 (38)	20.5 (30)	37.3 (150)	51.2 (111)
Other	12.5 (9)	12.5 (10)	3.3 (5)	5.6 (4)	11.7 (27)	15.8 (23)	8.7 (35)	3.7 (8)
None	0 (0)	0 (0)	1.3 (2)	0 (0)	0.4 (1)	0 (0)	1.2 (5)	0 (0)
**Education**								
Primary School or less	2.8 (2)	0 (0)	22.5 (34)	8.7 (6)	45.9 (105)	25 (36)	21.8 (87)	6.6 (14)
High/Secondary	36.1 (26)	75 (57)	23.8 (36)	75.4 (52)	32.8 (75)	54.9 (79)	34.3 (137)	58.7 (125)
Voc/Tech college	18.1 (13)	11.8 (9)	12.6 (19)	8.7 (6)	6.1 (14)	12.5 (18)	13.0 (52)	28. (61)
University	43.1 (31)	13.2 (10)	41.1 (62)	7.2 (5)	15.3 (35)	7.6 (11)	31.0 (124)	6.1 (13)
**Money earned by**								
Subsistence	1.5 (1)	13 (1)	4.9 (7)	9.4 (6)	15.5 (35)	14.1 (19)	9.0 (35)	5.3 (11)
Formal employment	37.3 (25)	27.3 (21)	38.7 (55)	14.1 (9)	51.3 (116)	43.7 (59)	61.5 (205)	57.4 (120)
Dependent on family	46.3 (31)	70.1 (54)	51.4 (73)	70.3 (45)	29.2 (66)	41.5 (56)	95.4 (132)	34.4 (72)
Student scholarship	14.9 (10)	1.3 (1)	4.9 (7)	6.3 (4)	4.0 (9)	0.7 (1)	4.6 (18)	2.9 (6)
**Number of wives#**								
0	70.8 (51)		73.7 (112)		59.4 (136)		56.5 (227)	
1	29.2 (21)	66.6 (10)	25.7 (39)	66.6 (6)	37.1 (85)	82.5 (47)	31.3 (126)	67.7 (63)
More than 1	0 (0)	33.3 (5)	0.7 (1)	33.3 (3)	3.5 (8)	17.5 (10)	12.3 (49)	32.3 (30)
**Number of Children**								
0	73.6 (53)	75 (60)	76.3 (116)	80.6 (58)	62.0 (142)	82.5 (66)	576 (230)	50 (109)
1	4.2 (3)	6.3 (5)	7.2 (11)	1.4 (1)	5.7 (13)	11.0 (16)	7.3 (29)	12.4 (27)
More than 1	22.2 (16)	18.7 (15)	16.4 (25)	18 (13)	32.3 (74)	43.5 (66)	35.1 (140)	37.6 (82)

^^^ Totals are not the same for all characteristics. Some participants did not answer all questions.

^#^ For females - number of wives their husband is married to.

### Prevalence of foreskin cuts

Ninety-nine percent of men (854/861) provided data on foreskin cutting: 10% (n = 87) reported a circumferential cut; 47% (n = 398) reported some form of longitudinal cut, and 43% (n = 369) no cut at all. The cutting varied significantly with age, education and region of origin (Table 2). Longitudinal cut was most frequent in men from Momase (58% of all men from that region); circumferential cut most frequent in men from New Guinea Islands (24%) and an uncut foreskin was most frequent in men from the Southern (50%) and Highlands (47%) regions (p < 0.001) (Table 3). Five variations or styles of longitudinal cut were recorded and varied by region of origin (Table 3).

**Table 2:** Demographic characteristics by foreskin cutting type

	**Uncut % (n)**		**Longitudinal cut % (n)**		**Circumferential cut % (n)**		**All men (n)**	**p-value**
**Overall Sample**	43.2 (369)		46.6 (398)		10.2 (87)		854	<.001
**Age**								
Under 25	34.8 (136)		55.5 (217)		9.7 (38)		391	<.001
25 and 0ver	53.0 (210)		36.6 (145)		10.4 (41)		396	
**Site**								
DWU	37.7 (78)		49.8 (103)		12.6 (26)		207	<.001
PAU	31.3 (62)		53.0 (105)		15.7 (31)		198	
Porgera	58.6 (133)		34.8 (79)		6.6 (15)		227	
Popondetta	43.2 (96)		50.0 (111)		6.8 (15)		222	
**Region**								
New Guinea Islands	26.5 (18)		50.0 (34)		23.5 (16)		68	<.001
Highlands	47.4 (190)		44.1 (177)		8.5 (34)		401	
Momase	28.8 (44)		58.2 (89)		13.1 (20)		153	
Southern	50.4 (115)		42.1 (96)		7.5 (17)		228	
**Marital Status**								
Single	35.1 (180)		54.0 (277)		10.9 (56)		513	<.001
Married	56.5 (183)		34.3 (111)		9.3 (30)		324	
Separated/Divorced	35.3 (6)		58.8 (10)		5.9 (1)		17	
**Religion**								
Anglican	47.8 (44)		45.7 (42)		6.5 (6)		92	<.05
Catholic	48.4 (62)		42.2 (54)		9.4 (12)		128	
Lutheran	40.2 (41)		47.1 (48)		12.7 (13)		102	
Pentecostal	42.9 (81)		49.2 (93)		7.9 (15)		189	
Seventh day Adventist	35.5 (92)		50.6 (131)		13.9 (36)		259	
Other	58.1 (43)		35.1 (26)		6.8 (5)		74	
None	62.5 (5)		37.5 (3)		0.0 (0)		8	
**Education**								
Primary School or less	52.8 (121)		42.8 (98)		4.4 (10)		229	<.001
High/Secondary	45.2 (122)		44.4 (120)		10.4 (28)		270	
Vocational/College	38.1 (37)		48.5 (47)		13.4 (13)		97	
University	34.4 (87)		51.4 (130)		14.2 (36)		253	
**Money earned by**								
Subsistence	37.2 (29)		47.4 (37)		15.4 (12)		78	<.001
Formal employment	51.9 (208)		38.2 (153)		10.0 (40)		401	
Dependent on family	37.1 (111)		53.2 (159)		9.7 (29)		299	
Student Scholarship	25.0 (11)		63.6 (28)		11.4 (5)		44	
**Number of Wives**								
0	35.1 (184)		54.0 (283)		10.9 (57)		524	<.001
1	51.9 (140)		37.4 (101)		10.7 (29)		270	
>1	74.1 (43)		24.1 (14)		1.7 (1)		58	
**Number of Children**								
0	35.8 (193)		53.4 (288)		10.8 (58)		539	<.001
1	44.6 (25)		44.6 (25)		10.7 (6)		56	
>1	57.5 (146)		33.5 (85)		9.1 (23)		254	

**Table 3:** Foreskin cutting classification by region of origin

	**New Guinea Islands**	**Highlands**	**Momase**	**Southern**	**ALL^**		***p *-value**
	***% (n)***	***% (n)***	***% (n)***	***% (n)***	***% (n)***		
***Overall foreskin cutting classification***							
**Uncut**	26 (18)	47 (190)	29 (44)	50 (115)	43 (369)		< .001
**Longitudinal cut**	50 (34)	44 (177)	58 (89)	42 (96)	47 (398)		
**Circumferential cut**	24 (16)	9 (34)	13 (20)	7 (17)	10 (87)		

***Longitudinal foreskin cut variations***							
**Longitudinal Cut: Variation (i)**	19 (13)	16 (66)	20 (31)	16 (37)	17 (147)		
Foreskin has been cut but still partially covers the head of the penis							
**Longitudinal Cut: Variation (ii)**	25 (17)	24 (96)	32 (49)	20 (46)	25 (208)		
Foreskin has been cut and remains loose behind the head of the penis							
**Longitudinal Cut: Variation (ii)**	2 (1)	2 (6)	3 (5)	2 (5)	2 (17)		
Foreskin has been cut on both sides leaving two or more tags							
**Longitudinal Cut: Variation (iv)**	3 (2)	1 (3)	2 (3)	1 (2)	1 (10)		
Foreskin has been cut with scarring along the penis							
**Longitudinal Cut: Variation (v)**	2 (1)	2 (6)	1 (1)	3 (6)	2 (14)		
‘Cowboy cut’ where foreskin can be pulled back over the head of the penis							

^^^ Totals in the ALL column for uncut and longitudinal cut classifications differ from the sum of regions because some participants did not provide information on region of origin.

### Circumstances of foreskin cutting

**Figure 1:**
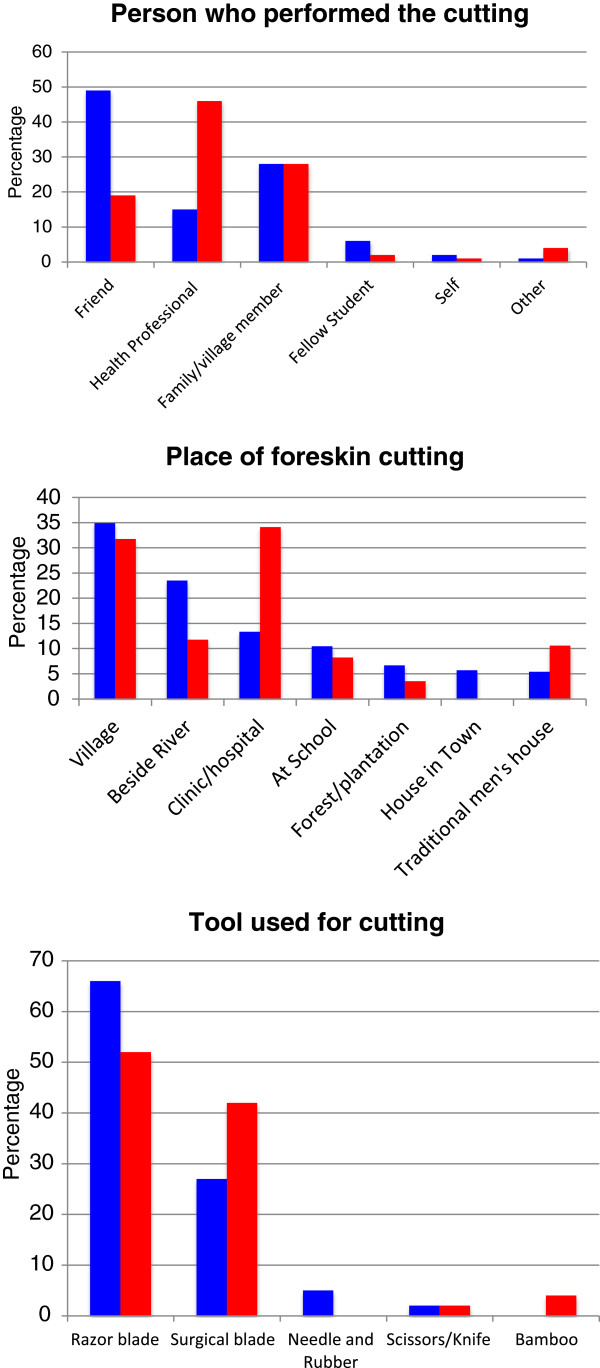
Place, person and tool used for foreskin cutting for men with longitudinal cut (in blue) and circumferential cut (in red).

Most cut men (82% longitudinal cut men; 72% circumferential cut men) reported having their foreskin cut between the ages of 10 and 20, with mean age 17 years (SD 4.77; Range 2–38) at longitudinal and 15 years (SD 6.66; Range 1–30) at circumferential cut (p < 0.001). The place and person performing the cuts varied significantly between longitudinal and round cut (p < 0.001). Longitudinal cuts were most frequently done in the village by a friend. Round cuts were most frequently done in a clinic by a health professional. Razors and surgical blades were the most utilized tools in all cuts (>90%), with scissors or bamboo used for 6% of circumferential cuts and a needle and rubber used for 5% of longitudinal cuts. For more details on place, person and tool used for foreskin cutting see Figure 1.

### Attitudes and beliefs about foreskin cutting

Attitudes and beliefs about foreskin cutting were investigated with both men and women around five thematic areas: (i) foreskin cutting and socio-cultural practice; (ii) foreskin cutting and sexual practice; (iii) foreskin cutting and sexual health; (iv) safety of foreskin cutting; (v) foreskin cutting and socio-cultural belief. Statements that participants responded to (yes, no, unsure) in each theme were deliberately mixed on the original questionnaire, however results are presented here in the five thematic areas. Responses to statements by uncut men, longitudinal cut men, circumferential cut men, all men and all women are given in Table 4.

**Table 4:** Attitudes and beliefs about foreskin cutting

		**Uncut men % (n)**	**Longitudinal cut men % (n)**	**Circumferential cut men % (n)**	***p *–value men**	**All men % (n) **	**Women % (n)**	**p-value men and women**
**Theme (i) Foreskin cutting and socio-cultural practice**								
Having a split foreskin is a part of my culture	Yes	9% (32)	25% (97)	24% (21)	< .001	18% (150)	8% (37)	< .001
	No	68% (246)	57% (222)	60% (52)		62% (520)	42% (204)	
	Unsure	23% (85)	19% (73)	16% (14)		20% (172)	51% (248)	
Having a round cut is part of my culture	Yes	8% (28)	15% (58)	38% (33)	< .001	14% (119)	10.5% (51)	< .001
	No	69% (247)	63% (245)	47% (41)		64% (533)	37% (182)	
	Unsure	23% (83)	22% (86)	15% (13)		22% (182)	52.5% (256)	
Having a split foreskin proves manhood	Yes	25% (90)	51% (200)	34% (29)	< .001	38% (319)	18% (80)	< .001
	No	32% (114)	19% (72)	30% (26)		26% (212)	18% (79)	
	Unsure	43% (153)	30% (116)	36% (31)		36% (300)	63% (274)	
Having a round cut proves manhood	Yes	24% (87)	44% (173)	51% (44)	< .001	36% (304)	22% (107)	< .001
	No	31% (113)	19% (74)	23% (20)		25% (207)	18% (87)	
	Unsure	45% (161)	37% (142)	26% (23)		39% (326)	60% (288)	
Men with a split foreskin are respected by their peers	Yes	18% (65)	35% (135)	29% (25)	< .001	27% (225)	7% (35)	< .001
	No	29% (107)	32% (124)	40% (34)		32% (265)	11% (53)	
	Unsure	53% (191)	33% (130)	31% (27)		41% (348)	82% (396)	
Men with a round cut are respected by their peers	Yes	17% (61)	31% (122)	42% (36)	< .001	26% (219)	11.5% (56)	< .001
	No	28% (102)	31% (120)	32% (28)		30% (250)	11% (55)	
	Unsure	55% (198)	38% (148)	26% (23)		44% (360)	77.5% (378)	
Having a split foreskin is forbidden by my religion	Yes	23% (82)	15% (59)	12% (10)	< .001	18% (151)	13% (63)	< .001
	No	42% (122)	52% (205)	55% (47)		45% (374)	28% (137)	
	Unsure	44% (158)	33% (127)	34% (29)		37% (314)	59% (284)	
Having a round cut is forbidden by my religion	Yes	22% (79)	15% (59)	9% (8)	< .001	18% (146)	13% (62)	< .001
	No	35% (125)	51% (199)	64% (55)		45% (379)	29% (141)	
	Unsure	43% (157)	34% (131)	27% (23)		37% (311)	58% (276)	
Having a split foreskin is forbidden by my custom/culture	Yes	18% (63)	13% (50)	14% (12)	< .001	15% (125)	11.5% (54)	< .001
	No	44% (156)	65% (250)	64% (55)		56% (461)	30% (145)	
	Unsure	39% (139)	22% (86)	22% (19)		29% (244)	58.5% (281)	
Having a round cut is forbidden by my custom/culture	Yes	19% (68)	15% (58)	11% (10)	< .001	16% (136)	11.5% (55)	< .001
	No	43% (157)	61% (236)	70% (61)		54% (454)	30% (145)	
	Unsure	38% (136)	24% (95)	18% (16)		30% (247)	58.5% (280)	
**Theme (ii) Foreskin cutting and sexual practice**								
A split foreskin decreases sexual pleasure for a man	Yes	13% (47)	16% (62)	8% (7)	< .001	14% (116)	9% (46)	< .001
	No	26% (95)	50% (194)	50% (42)		39% (331)	22% (104)	
	Unsure	61% (220)	34% (135)	43% (37)		47% (392)	69% (330)	
A round cut decreases sexual pleasure for a man	Yes	14% (51)	14% (56)	15% (13)	< .001	14% (120)	10% (48)	< .001
	No	26% (93)	43% (166)	53% (46)		36% (305)	22% (104)	
	Unsure	60% (218)	43% (168)	32% (28)		49% (414)	68% (329)	
Sex lasts longer for men who have a split foreskin	Yes	19% (68)	44% (171)	22% (19)	< .001	31% (258)	9.5% (46)	< .001
	No	12% (42)	13% (52)	20% (17)		13% (111)	10.5% (51)	
	Unsure	69% (250)	43% (166)	58% (50)		56% (466)	80% (387)	
Sex lasts longer for men who have a round cut	Yes	19% (67)	38% (145)	47% (41)	< .001	30% (253)	13% (63)	< .001
	No	1% (41)	12% (46)	14% (12)		12% (99)	8.5% (40)	
	Unsure	70% (252)	50% (195)	39% (34)		58% (481)	78.5% (375)	
Having a split foreskin encourages men to have more sexual partners	Yes	28% (100)	41% (159)	25% (21)	< .001	34% (280)	17% (82)	< .001
	No	19% (70)	30% (116)	44% (37)		27% (223)	21.5% (102)	
	Unsure	53% (192)	29% (113)	31% (26)		40% (331)	61.5% (294)	
Having a round cut encourages men to have more sexual partners	Yes	27% (99)	40% (153)	26% (22)	< .001	33% (274)	19% (90)	< .001
	No	18% (67)	26% (102)	48% (41)		25% (210)	21% (100)	
	Unsure	54% (196)	34% (132)	27% (23)		42% (351)	60% (281)	
Women prefer to have sex with a man who has a split foreskin	Yes	31% (112)	51% (200)	39% (34)	< .001	41% (346)	15% (72)	< .001
	No	10% (35)	7% (26)	11% (9)		8% (70)	11% (52)	
	Unsure	59% (214)	42% (165)	50% (43)		50% (422)	74% (358)	
Women prefer to have sex with a man who has a round cut	Yes	31% (114)	49% (193)	50% (44)	< .001	42% (351)	22% (106)	< .001
	No	10% (35)	6% (25)	8% (7)		8% (67)	8% (38)	
	Unsure	59% (213)	45% (175)	34% (37)		50% (425)	70% (338)	
**Theme (iii) foreskin cutting and sexual health**								
Men with a split foreskin do not need to use condoms to protect them from STIs & HIV	Yes	13% (45)	17% (65)	11% (9)	< .001	14% (119)	10% (48)	< .001
	No	50% (181)	61% (239)	69% (59)		57% (479)	50% (242)	
	Unsure	37% (135)	22% (88)	20% (17)		29% (240)	40% (195)	
Men with a round cut do not need to use condoms to protect them from STIs & HIV	Yes	14% (52)	17% 965)	9% (8)	< .001	15% 9125)	10% (47)	< .001
	No	49% (176)	61% (237)	72% (61)		57% (474)	51% (246)	
	Unsure	37% (132)	23% (89)	19% (16)		28% (237)	39% (190)	
Men with a split foreskin can become infected with HIV	Yes	59% (211)	64% (251)	65% (56)	.015	63% (518)	45% (219)	< .001
	No	6% (20)	10% (39)	9% (8)		8% (67)	10% (50)	
	Unsure	36% (127)	26% (100)	26% (22)		30% (249)	44% (213)	
Men with a round cut can become infected with HIV	Yes	58% (209)	64% (250)	68% (60)	.059	62% (519)	41% (198)	< .001
	No	7% (26)	9% (36)	10% (9)		8% (71)	13% (64)	
	Unsure	35% (126)	27% (106)	22% (19)		30% (251)	46% (220)	
A split foreskin reduces the risk of becoming infected with HIV	Yes	31% (112)	37% (145)	33% (28)	.324	34% (285)	27% (130)	< .001
	No	25% (92)	26% (101)	28% (24)		26% (217)	22% (105)	
	Unsure	44% (157)	37% (142)	39% (34)		40% (333)	51% (248)	
A round cut reduces the risk of becoming infected with HIV	Yes	35% (126)	39% (153)	36% (32)	.813	37% (311)	33.5% (162)	< .001
	No	26% (93)	25% (98)	24% (21)		25% (212)	17% (82)	
	Unsure	39% (141)	36% (141)	40% (35)		38% (317)	49.5% (239)	
**Theme (iv) foreskin cutting and safety**								
It is safe to use the same blade or razor to split or remove the foreskin of many men at one time	Yes	5% (17)	8% (30)	6% (5)	< .001	6% (52)	3% (16)	< .001
	No	81% (293)	87% (339)	89% (77)		85% (709)	75% (365)	
	Unsure	14% (51)	5% (21)	5% (4)		9% (76)	21% (104)	
Splitting the foreskin in a village by a friend or relative is a safe procedure	Yes	8% (27)	15% (60)	17% (15)	.005	12% (102)	5.5% (26)	< .001
	No	67% (237)	64% (245)	65% (55)		65% (537)	63.5% (297)	
	Unsure	26% (91)	21% (80)	18% (15)		23% (186)	31% (146)	
Removing the foreskin in a village by a friend or relative is a safe procedure	Yes	8% (27)	12% (46)	17% (15)	.043	11% (88)	6% (30)	< .001
	No	67% (243)	66% (258)	66% (57)		64% (558)	61.5% (296)	
	Unsure	25% (92)	22% (87)	17% (15)		23% (194)	32.5% (157)	
Having a round cut by a doctor or nurse in a clinic or hospital is a safe procedure	Yes	83% (298)	92% (360)	88% (77)	.005	88% (735)	71% (342)	< .001
	No	3% (10)	2% (7)	3% (3)		2% (20)	5% (23)	
	Unsure	14% (51)	6% (25)	8% (7)		10% (83)	24% (118)	
**Theme (v) foreskin cutting and socio-cultural beliefs**								
Allowing blood to flow when the foreskin is split or removed is important in my custom/culture	Yes	21% (76)	26% (102)	30% (26)	.067	24% (204)	7% (33)	< .001
	No	42% (151)	46% (180)	38% (33)		43% (364)	25% (121)	
	Unsure	40% (133)	28% (111)	32% (28)		32% (272)	68% (326)	
A man needs to eat special food in the days after having his foreskin split or removed	Yes	46% (166)	71% (278)	61% (53)	< .001	59% (497)	25% (123)	< .001
	No	7% (26)	11% (42)	18% (16)		10% (84)	10% (47)	
	Unsure	47% (170)	18% (70)	21% (18)		31% (258)	65% (311)	
A man needs to reduce the amount of water he drinks in the days after having his foreskin split or removed	Yes	52% (188)	85% (333)	72% (63)	< .001	69% (584)	22% (107)	< .001
	No	7% (26)	5% (19)	13% (11)		7% (56)	10% (49)	
	Unsure	41% (148)	10% (41)	15% (13)		24% (202)	68% (327)	
A man needs to avoid women in the days after having his foreskin split or removed	Yes	68% (245)	91% (355)	90% (78)	< .001	81% (678)	48% (230)	< .001
	No	4% (16)	3% (10)	2% (2)		3% (28)	4% (18)	
	Unsure	28% (101)	7% (27)	8% (7)		16% (135)	48% (232)	
A split foreskin makes a man’s body grow strong	Yes	30% (108)	54% (213)	43% (37)	< .001	43% (358)	16% (76)	< .001
	No	11% (39)	9% (36)	16% (14)		11% (89)	12% (56)	
	Unsure	59% (210)	37% (144)	41% (35)		46% (389)	72% (349)	
A round cut makes a man’s body grow strong	Yes	31% (111)	53% (209)	50% (44)	.001	43% (364)	18% (86)	< .001
	No	9% (33)	7% (27)	14% (12)		9% (72)	11% (52)	
	Unsure	60% (217)	40% (156)	36% (32)		48% (405)	71% (343)	
A split foreskin makes the penis grow bigger	Yes	31% (111)	53% (206)	37% (32)	< .001	42% (349)	11% (52)	< .001
	No	9% (33)	14% (55)	19% (16)		12% (104)	13% (61)	
	Unsure	60% (217)	33% (131)	44% (38)		46% (386)	76% (369)	
A round cut makes the penis grow bigger	Yes	31% (112)	48% (189)	42% (37)	< .001	40% (338)	14% (68)	< .001
	No	10% (35)	14% (54)	22% (19)		13% (108)	11.5% (55)	
	Unsure	59% (214)	38% (149)	36% (32)		47% (395)	74.5% (358)	

Key findings for **(i) Foreskin cutting and socio-cultural practice**: Most men (63%) stated that foreskin cutting was not a part of their cultural practice/tradition. For women 42% stated it was not their cultural practice/tradition, although half (51%) were unsure. Around a third of men and less than one in five women agreed that having a cut foreskin proves manhood. Less than 20% of men and women stated that foreskin cutting was forbidden by their custom/tradition or by their religion.

Key findings for **(ii) foreskin cutting and sexual practice**: A third of men and around 20% of women agreed that having a cut foreskin encourages men to have more sexual partners. Less than 15% of men and 10% of women agreed that having a cut foreskin decreases sexual pleasure for men with about half of men and the majority of women responding they were unsure. Around a third of men and one in ten women agreed to the statement that sex lasts longer for men with a cut foreskin. More than 40% of men but less than 20% of women agreed to the statement that women prefer to have sex with man with cut foreskin; half of men and almost three-quarters of women responded they were unsure.

Key findings for **(iii) foreskin cutting and sexual health**: More than half of men and a little under half of women agreed that men with a cut foreskin can become infected with HIV. Around a third of men and women agreed that having a cut foreskin reduces the risk of becoming infected with HIV. The majority of both men (57%) and women (51%) disagreed with the statement that men with a cut foreskin do not need to use condoms to protect from STI and HIV.

Key findings for **(iv) safety of foreskin cutting**: Around 10% of men and 5% of women agreed that foreskin cutting in a village by a friend or relative was a safe procedure. Almost all men and three quarters of women disagreed that it is safe to use the same blade or razor to cut the foreskin of many men at one time. Most men (88%) and women (71%) agreed that having a foreskin cut by a health professional in a health facility is a safe procedure.

Key findings for **(v) foreskin cutting and socio-cultural beliefs**: A quarter of men agreed that allowing the blood to flow when the foreskin is cut is important in their culture/custom. The majority of men agreed that it was important to eat special food and to reduce the amount of water in the days following the cut. More than 80% of men agreed that men need to stay away from women after having a foreskin cut. Around 40% of men agreed that a cut foreskin makes a man’s body grow strong and the penis grow bigger; less than 20% women agreed, with the majority unsure.

### Foreskin cutting and sexual practice

Men with circumferential cut had significantly fewer lifetime female sexual partners (median 5) compared to the men with longitudinal cut (6) or uncut men (7) (p < 0.05). There was no difference in condom use at last female sex between circumferential cut, longitudinal cut and uncut men (35%, 32% and 33% respectively; p = 0.9).

### Acceptability of male circumcision

Most uncut men and longitudinal cut men stated they would remove their foreskin or its remnant part, if it reduced the risk of HIV infection (71% and 76%) or if it had an overall health benefit (84% and 88%) (Table 5). The vast majority would prefer the procedure done in a formal health facility by a health worker. Almost two-thirds (64%) of uncut men and half (51%) of longitudinal cut men stated they were planning to have their foreskin removed at some time in the future (Table 5).

**Table 5:** Acceptability of male circumcision for self and male child for uncut, longitudinal cut and round cut men

		**Uncut men**	**Longitudinal cut men**	**Round cut men**	**p-value**
		**% (n)^**	**% (n)**	**% (n)**	
**Would have foreskin completely removed if it had a health benefit**	Yes	76 (269)	88 (278)		<.001
	Maybe	12 (45)	0		
	No	12 (42)	12 (38)		
**Would have foreskin completely removed if it reduced the risk of getting HIV**	Yes	71 (250)	84 (258)		<.001
	Maybe	13 (46)	0		
	No	15(55)	16 (49)		
**Preferred place to have foreskin removed**	Hospital/clinic	95 (312)	97 (247)		.26
	Others	3 (9)	2 (5)		
	Not sure	2 (8)	1 (2)		
**Preferred person to remove foreskin**	Health worker	90 (298)	95 (292)		<.01
	Other	4 (14)	3 (10)		
	Don’t know /not want MC	6 (19)	1 (4)		
**Planning to remove foreskin**	Yes	64 (229)	51 (163)		<.001
	Maybe	14 (50)	26 (84)		
	No	22 (78)	22 (70)		
**Recommend foreskin removal to friends**	Yes	64 (210)	64 (209)	89 (57)	<.001
	No	36 (116)	36 (116)	11 (7)	
**Would have the foreskin removed from male child if it had a health benefit**	Yes	86 (260)	92 (280)	95 (58)	<.05
	No	14 (43)	8 (26)	5 (3)	
**Would have the foreskin removed from male child if it reduced the risk of HIV or STIs**	Yes	87 (250)	93 (285)	91 (53)	<.05
	No	13 (38)	7(20)	9 (5)	

^^^ Totals are not the same for all characteristics. Some participants did not answer all questions.

Almost all men and three-quarters of women (74%) stated they would remove the foreskin of their male child if it reduced the risk of HIV infection, and even higher proportions if it had an overall health benefit (Table 5).

## Discussion

This is the first study conducted in PNG that combines the investigation of prevalence, beliefs, attitudes and practices about foreskin cutting and the acceptability of male circumcision. It thus addresses a vital area for public health and HIV prevention in the country. Results expand the evidence vital for the National AIDS Council, National Department of Health and other policy makers to more effectively plan HIV prevention strategies.

The overall prevalence of foreskin cutting in the study was 57%, however only 10% of men reported the complete removal of the foreskin, the procedure on which international HIV prevention strategies and recommendations are based upon. The prevalence of foreskin cutting in this study was higher than the 25.8% longitudinal cut and 3.4% circumferential cut documented in recent studies in plantation workers in the Highlands region, but similar to studies in the national capital [61-63,65]. The deliberate approach to include participants from diverse geographic locations and cultural backgrounds allowed a sample with a similar regional proportionality to the PNG population. Although this does not automatically imply representativeness or generalisability across the PNG population, this characteristic does enable closer analysis of the diversity of opinions and experience. The highest prevalence of both longitudinal and circumferential cuts in this study were from men from New Guinea Islands and Momase. This was not surprising given the numerous cultural groups with a tradition of foreskin cutting in these regions. However around half of men from the Highlands and Southern regions also reported having a cut foreskin (most often longitudinal cut). This was surprising given there are far fewer cultural groups with a tradition of foreskin cutting in these regions. Older men from Highlands and Southern regions were less likely to have a foreskin cut, or have detailed knowledge about foreskin cutting. This reflects recent rapid changes that enable modern education, travel and work practices that facilitate the exchange of knowledge, beliefs and practices, including foreskin cutting.

**Figure 2:**
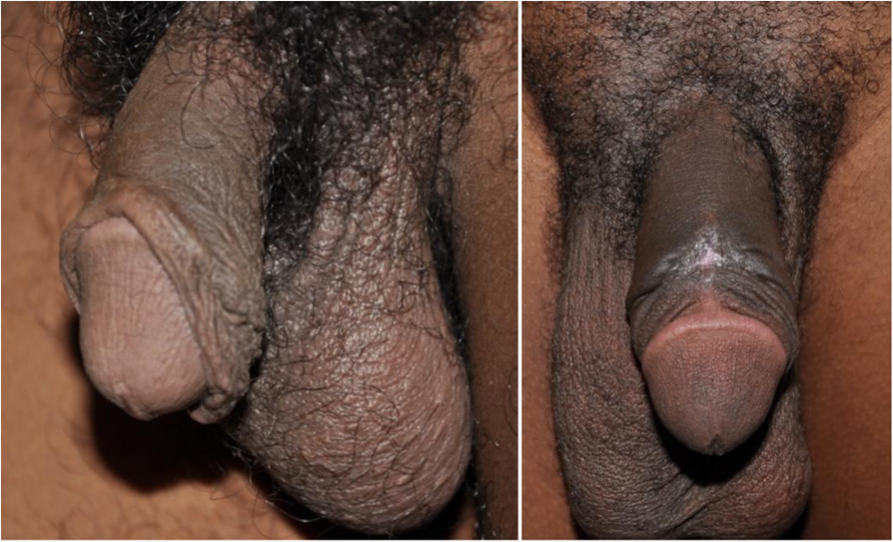
Longitudinal Foreskin cut: Variant (i) Foreskin cut but still partially covers the glans penis; Variant (ii) Foreskin cut and remains loose behind the glans penis.

This study provides further evidence that longitudinal cuts are the major forms of foreskin cutting in PNG[54,57,66,67]. Of men with dorsal longitudinal cuts in this study, more than half reported a variant where the glans penis is totally exposed. In this variant, the remnant foreskin hangs loosely on the ventral surface of the penis permanently exposing the inner surface of the foreskin. The foreskin then reduces in size, becomes dry and is visually similar to the outer surface of the foreskin. In some men with this variant, the foreskin reduces to such an extent that the penis appears very similar to a circumcised penis (See Figure 2). It is unclear what the implications of such longitudinal cuts are for HIV prevention. The major explanation of how MC protects against HIV is that the inner aspect of the foreskin is the prime site for HIV entry on the penis. The inner aspect (and the frenulum) have a thinner keratin layer than the glans or penile shaft and so enable HIV entry to Langerhans cells which carry specific HIV receptors [68]. Removing the foreskin through MC removes the prime site for HIV entry and so reduces the risk of HIV infection. This leads to the potential that changes to the exposed inner surface and reduction in the surface area of the remnant foreskin may provide some protection against HIV infection for men with this variant. However the potential protective effect is currently unknown [57,69]. A concerted research effort to investigate the potential protective effects of longitudinal cuts for HIV acquisition in PNG is essential given the scale of longitudinal cuts documented. This is particularly important given that most of the foreskin cutting does not occur within traditional initiation rituals or within the formal health sector, but predominantly between peers as an evolving contemporary socio-cultural practice. Moreover, many men with longitudinal cuts in PNG consider themselves as ‘circumcised’ because of the appearance of their penis and because the commonly used *Tok Pisin* terms *katim skin bilong kok* or *katim kok* do not differentiate between longitudinally cut or totally removed foreskin [46]. Studies from PNG, Rwanda, Swaziland and Kenya have highlighted regional and cultural variations of foreskin cutting that do not completely remove the foreskin, and that many of these men consider themselves ‘circumcised’. This re-enforces the need for further research and more nuanced understanding of MC for HIV prevention in such settings [33,57,70,71]. This is particularly important given cultural contexts are constantly evolving and adapting to new influences and circumstances [72].

MC for adult men was viewed positively. Three quarters of uncut men (76%) stated that they would remove their foreskin if it had a health benefit and 71% if it reduced the risk of HIV infection. These results reflect similarly high proportions (86%) of male university students willing to have the foreskin removed in a pilot study [65] and qualitative findings showing PNG men to be generally in favour of MC for HIV prevention [46]. Further, men with an existing longitudinal cut were overwhelmingly in favour of having the remnant foreskin removed if it reduced the risk of HIV infection. Most of these men had their longitudinal cut in a community setting, but almost all stated they would prefer to have the removal procedure conducted in a health facility by a health professional which they considered a safer option. The high proportion of men willing to have MC is similar to studies in high HIV prevalence settings in Africa (median 65%) but somewhat greater than studies in other moderate prevalence settings, such as India (58%), Thailand (14% and 25% before and after information) and Dominican Republic (29% and 67% before and after information) [13,40,41,43]. Methodology, study population, HIV prevalence and socio-cultural context are so different across these studies that comparisons are hard to interpret. Moreover men in these studies were categorised in a circumcised – uncircumcised dichotomy, making interpretation even more difficult for the PNG context where the longitudinal cut is the major form of cutting. However, these studies do reflect the need to investigate understandings and cultural contexts of specific populations to inform if, or how, MC may be a part of the local response to HIV.

MC for children was viewed positively. More than 90% of men and three quarters of women stated they would remove the foreskin of their child if it had a health benefit and/or reduced the risk of HIV. These rates are similar to parents in some high prevalence African settings and provide some of the first evidence about parents’ willingness to circumcise their sons in a moderate HIV setting [13]. This provides a unique challenge to the PNG health system that struggles to provide even basic primary health care services for the majority of the population. There are only 0.5 doctors and 5 nurses/midwives per 10 000 people in PNG and only 39% of births are supervised by skilled birth attendants [73,74]. Having a large number of adults, adolescents or infants present for MC would require considerable training, infrastructure and resource allocation across the entire health system.

Specific attitudes and beliefs documented in this study have direct relevance for policy makers. When given the ability to respond yes, no, or unsure to statements about foreskin cutting it was common for around a third of men and often more than half of women responded ‘unsure’. Although this high level of uncertainly may be of concern to some service providers or policy makers, it reflects the dynamic socio-cultural context in PNG and that many people are aware of foreskin cutting but may not have fixed attitudes or beliefs towards foreskin cutting. This provides an opportunity for public health campaigns, as called for by Kelly et al. [46], to provide accurate information in a culturally sensitive way that builds on the strength of culture, rather than destroying it. In a country where one’s culture and religion is so explicitly referred to in everyday life, it is important to note that few men and women stated foreskin cutting was forbidden by their custom/tradition or religion. Further, none of the beliefs about culture, religion or sexual practice documented in this study contradict the positive views of men and parents towards MC, or the appropriateness of providing MC services in this setting.

It is pleasing to note that the majority of both men and women believe men with a cut (or removed) foreskin still need to use condoms to prevent STI and HIV. However, of concern is one quarter of men and 40% of women stated they were unsure. This requires current public health messages about STI and HIV prevention to emphasise that condoms are for everyone regardless of a man’s foreskin cutting status. It is also encouraging that almost all men and women think it is unsafe to use the same blade to cut many men. This is likely to be due to cultural beliefs about the contagion potential of blood re-enforced by public HIV prevention messages about the danger of sharing tools for body cutting, tattooing or scarification practices [75]. Nevertheless, 19% of men and 52% of women were not sure or do not think it is necessary to avoid sexual contact in the days after the foreskin cut, highlighting the need for more specific MC education.

As in other low-moderate HIV burden settings with heterosexual transmission, a key challenge is how to expand existing clinical MC services, increase the safety of existing community foreskin cutting practices and/or introduce new MC services appropriate to local contexts. Decision makers must consider programs that deliver the greatest epidemiological and public health impact while being responsive to diverse socio-cultural practices and health service capacity [33,45,46]. In this context, researchers and policy makers in PNG are actively considering international and local evidence to inform policy making and future research [76,77].

The major strength of this study was that it was a partnership between PNG and Australian universities and two large resource companies across four provinces at sites where people from across PNG come to study or work. This allowed access to a great diversity of men and women who provided data on traditional and contemporary foreskin cutting practices and beliefs. However, only some major descriptive analysis of this large quantitative data set on foreskin cutting practices and beliefs could be presented in this paper. Further more detailed analyses of quantitative data and findings from qualitative interviews and focus group discussions will be published separately. Given the hyper-diversity of having more than 800 languages, and thus diverse sets of beliefs and practices in the country, this study provides a limited, although valuable snapshot of current foreskin cutting and the acceptability of MC in PNG.

## Conclusion

This study considerably expands the evidence base of current foreskin cutting practices in this moderate HIV prevalence setting. The major form of foreskin cut is the longitudinal cut along the dorsal surface resulting in the remnant foreskin hanging from the ventral surface of the penis. In most cases this totally exposes the glans penis and results in a remnant foreskin that is dry, reduced in size and with the inner surface visually similar to the outer surface. Foreskin removal (MC), from both uncut men and men with an existing longitudinal cut was considered appropriate and acceptable by most men and women in this study. Potential MC services will need to be responsive to the great diversity of local socio-cultural beliefs and practices and existing health service constraints. Research evidence of the protection conferred by longitudinal cuts is urgently needed to inform HIV prevention strategies in this setting. This study provides vital evidence on current foreskin cutting beliefs and practices and the implications for the acceptability of MC for HIV prevention in PNG. It thus enables more effective planning of HIV prevention in PNG and other populations with dynamic and varied socio-cultural foreskin cutting practices.

## Competing interests

The authors declare there they have no competing interests.

## Authors’ contributions

DM and WJM conceived, designed and coordinated the study and drafted the manuscript. RT, TM, CM, FF, MRM, MW, KB participated in the design and coordination of the study; collected, managed and analysed data; helped draft and revise the manuscript. RM and JK managed, analysed and interpreted data and help to draft and revise the manuscript. All authors have read and approved the final manuscript.

## Pre-publication history

The pre-publication history for this paper can be accessed here:

http://www.biomedcentral.com/1471-2458/13/818/prepub

## Supplementary Material

Additional file 1: Figure S1 Male Questionnaire in English. Click here for file

Additional file 2: Figure S2 Female Questionnaire in English. Click here for file
