# Supplementary material for: Foreskin cutting beliefs and practices and the acceptability of male circumcision for HIV prevention in Papua New Guinea
Source: BMC Public Health. 2013 Sep 9;13:818. doi: 10.1186/1471-2458-13-818 (PMC3846639; doi:10.1186/1471-2458-13-818)
Supplement: Additional file 1: Figure S1 — Male Questionnaire in English. [file 1471-2458-13-818-S1.pdf]

Acceptability of Male Circumcision for HIV Prevention in PNG Study

A Joint JCU, PAU, DWU Study 2010-2012: NHMRC Project Grant 601003

Questionnaire for Male Participants

Participant Number

You are invited to take part in a research project that is seeking to find out if increasing the number of men who are circumcised would be an acceptable way to reduce the spread of HIV in Papua New Guinea (PNG). This is important because it has been shown in some other countries that men who are circumcised are less likely to contract HIV than men who are not circumcised. The study will ask both men and women 18 years and over about their experience and attitudes about male circumcision. Your participation in the study as a man will help us understand the possible acceptability of male circumcision for HIV prevention in PNG. The study is being conducted by **Dr David MacLaren** from **James Cook University**, in partnership with researchers from **Pacific Adventist University (PAU)** and **Divine Word University (DWU)**.

You are invited to complete this self-administered questionnaire. Questions in it are specifically for men and ask about your thoughts about HIV, and your experiences and opinions of male circumcision. The questionnaire will take between 30 – 60 minutes to complete. By completing and returning the questionnaire you are giving consent for the results to be used in this study. The survey is anonymous and your name will not be asked in the survey. On the last page of the questionnaire there is an invitation and instructions about how to progress to the next stage of the study. This will involve seeing a health professional who will examine your genitals to see if you have had a circumcision and the type of circumcision you may have had. In some cases the health professional will ask to take a photo of your genitals to record different types of circumcision.

Do I have to take part of this study?
Taking part in this study is completely voluntary and you can stop taking part in the study at any stage without explanation or prejudice. You may also withdraw any data from the study before it is analysed.

This is a very personal subject: What if I become distressed?
As this study is about sexual health and is of a sensitive nature, some people may find some of the questions a little distressing. If you do feel upset or distressed in any way, please advise the researcher or the contacts at the bottom of this sheet and you will be referred to someone who can help you.

What will happen to the results of the study?
Your responses and identity will be kept strictly confidential. The data from the study will be used in research publications and reports to PNG Ministry of Heath, PNG National and Provincial AIDS Councils, other health researchers and health service providers. You will not be identified in any way in these publications.

If you have any questions about the study, please contact the following:

Dr David MacLaren
James Cook University
Phone: +61 (7) 4042 1658
Email: david.maclaren@jcu.edu.au

Ms Rachael Tommbe
Pacific Adventist University
Phone:+675 328 0342
Email: Rachael.Tommbe@pau.ac.pg

Dr Clement Manineng
Divine Word University
Phone: +675 424 1887
Email: cmanineng@dwu.ac.pg

**Section 1: Background Characteristics**<br>In this first section of the questionnaire you will be asked some questions about your background.<br>Please circle the number beside the correct answer or complete the statement beside the question.				
**No.**	**Questions**			**Go To**
**1**	What is your gender	Male<br>Female	1<br>2	
**2**	How old are you?	I am ____________years old		
**3**	Which Province do you come from?<br>(circle more than one if required)	Bougainville (ARB)<br>Central<br>East New Britain<br>East Sepik<br>Eastern Highlands<br>Enga<br>Gulf<br>Hela<br>Jiwaka<br>Madang<br>Manus<br>Milne Bay<br>Morobe<br>NCD<br>New Ireland<br>Oro<br>Sandaun<br>Simbu<br>Southern Highlands<br>West New Britain<br>Western<br>Western Highlands<br>Other(specify)________________	1<br>2<br>3<br>4<br>5<br>6<br>7<br>8<br>9<br>10<br>11<br>12<br>13<br>14<br>15<br>16<br>17<br>18<br>19<br>20<br>21<br>22<br>23	
**4**	What is your religion?	Christian<br>Hindu<br>Muslim<br>None<br>Other ______________________	1 →<br>2 →<br>3 →<br>4 →<br>5 →	**Go to Q 5**<br><br>**All others Go to Q 6**
**5**	What church do you belong to?	Anglican<br>AOG<br>Baptist<br>Catholic<br>Christian Brethren Church<br>ECB<br>Evangelical Churches<br>Jehovah's Witness<br>Lutheran<br>Mormon<br>Pentecostal<br>PNG Bible Church<br>Revival<br>SDA<br>United<br>Other (specify)________________<br>None	1<br>2<br>3<br>4<br>5<br>6<br>7<br>8<br>9<br>10<br>11<br>12<br>13<br>14<br>15<br>16<br>17	
**6**	What is your current marital status?	Single<br>Married<br>Separated<br>Divorced<br>Widower<br>Other (specify)________________	1<br>2<br>3<br>4<br>5<br>6	

7	How many wives do you currently have?<br>(if not married mark '0')	I have ________________wives		
8	How many male children do you have?<br>(if no male children mark '0')	I have ____________male children		
9	How many female children do you have?<br>(in no female children mark '0')	I have __________female children		
10	What is the highest level of formal education you have completed?	Have not attended school<br>Elementary (prep – elementary 2)<br>Primary (grade 3-8)<br>High School (grade 9-10)<br>Secondary School (grade 11-12)<br>Vocational<br>Technical/College<br>University<br>Other (specify)________________	1<br>2<br>3<br>4<br>5<br>6<br>7<br>8<br>9	
11	Are you currently enrolled in a school, college or university?	Yes<br>No	1 →<br>2 →	**Go to 12**<br>**Go to 13**
12	What year level are you currently studying?	High School (grade 9-10)<br>Secondary School (grade 11-12)<br>Vocational/College Year 1<br>Vocational/College Year 2<br>Vocational/College Year 3<br>University Year 1<br>University Year 2<br>University Year 3<br>University Year 4<br>University Post-graduate<br>Other (specify)_______________	1<br>2<br>3<br>4<br>5<br>6<br>7<br>8<br>9<br>10<br>11	
13	What do you do to earn money?	Self employed e.g. grow crops<br>Manual work/field worker<br>Trade or technical work<br>Professional work<br>Dependant on family/guardian<br>Student Scholarship<br>Other (specify)________________	1<br>2<br>3<br>4<br>5<br>6<br>7	

Section 2: Knowledge of HIV

In this section of the questionnaire you will be asked a series of questions about HIV and how it is transmitted

14	How much do you know about HIV?	Nothing at all<br>A little<br>Moderate amount<br>A lot	1<br>2<br>3<br>4	
15	Where does your information about HIV come from?<br>(circle more than one if required)	Radio<br>TV<br>News paper<br>Non Government Organisation<br>Religious Organisation<br>Teacher<br>Friend<br>Relative<br>Doctor/Nurse/Health Worker<br>VCT Centre<br>Clinic/Health Centre<br>Pamphlet/brochure<br>Poster<br>Internet<br>Others (specify) ______________	1<br>2<br>3<br>4<br>5<br>6<br>7<br>8<br>9<br>10<br>11<br>12<br>13<br>14<br>15	
16	Can a woman get HIV from having sex with a man who has HIV?	Yes<br>No<br>Not sure	1<br>2<br>3	

**17**	Can a man get HIV from having sex with a woman who has HIV?	Yes<br>No<br>Not sure	1<br>2<br>3	
**18**	Can a man get HIV from having sex with a man who has HIV?	Yes<br>No<br>Not sure	1<br>2<br>3	
**19**	Can a person get HIV from a needle, razor or other cutting tools that have already been used by someone else for tattooing, scarification or circumcision?	Yes<br>No<br>Not sure	1<br>2<br>3	
**20**	Can a person get HIV from mosquito bites?	Yes<br>No<br>Not sure	1<br>2<br>3	
**21**	If someone with HIV coughs or sneezes near another person, can that person get HIV?	Yes<br>No<br>Not sure	1<br>2<br>3	
**22**	Can a person get HIV by hugging someone who has HIV?	Yes<br>No<br>Not sure	1<br>2<br>3	
**23**	Can a person get HIV by sharing food with someone who has HIV?	Yes<br>No<br>Not sure	1<br>2<br>3	
**24**	If a woman with HIV is pregnant, can her baby become infected with HIV?	Yes<br>No<br>Not sure	1<br>2<br>3	
**25**	Can the family planning pill protect a woman from HIV infection?	Yes<br>No<br>Not sure	1<br>2<br>3	
**26**	If condoms are used correctly during sex do they help protect people from getting HIV?	Yes<br>No<br>Not sure	1<br>2<br>3	
**27**	Can someone who looks healthy have HIV?	Yes<br>No<br>Not sure	1<br>2<br>3	

Section 3: History of Sexual Practices

In this section of the questionnaire you will be asked some very personal questions about sex. We appreciate your honesty and assure you that none of this information will be linked to you as an individual.

**28**	Have you ever had sex with anyone at any time in your life? By that we mean vaginal or anal penetrative sex.	Yes<br>No	1 →<br>2 →	**Go to 29**<br>**Go to 43**
**29**	How old were you when you first had sex?	I was ____________years old		
**30**	The first time you had sex, how old was the person you had sex with?<br>(If unsure of exact age please estimate)	The person was __________years old		
**31**	Have you ever used a condom with a sexual partner at any time in your life?	Yes<br>No	1<br>2	
**32**	How many *women* have you had sex with in your life?<br>(If unsure of exact number please estimate)	I have had sex with ___________women		
**33**	The *last* time you had sex with a *woman* did you use a condom?	Yes<br>No	1<br>2	
**34**	If yes, why did you use a condom the last time you had sex?<br>(circle more than one if required)	Avoid pregnancy<br>Avoid sexually transmitted infection/HIV<br>Partner's choice<br>Other (specify)______________________	1<br>2<br>3<br>4	

**35**	If No, why didn't you use a condom the last time you had sex?<br>(circle more than one if required)	Not available<br>Too expensive<br>Partner objected<br>Don't know how to use one<br>Do not like using them<br>I trust my partner<br>I was drunk/using drugs<br>Condoms reduce pleasure<br>Condom's don't work<br>Goes against my religion<br>I don't care if I use one or not<br>Other (specify)____________________	1<br>2<br>3<br>4<br>5<br>6<br>7<br>8<br>9<br>10<br>11<br>12	
**36**	Have you ever had anal sex with a *man*?	Yes<br>No	1 →<br>2 →	**Go to 37**<br>**Go to 41**
**37**	How many *men* have you had sex with in your life?<br>(If unsure please estimate)	I have had sex with __________men		
**38**	The *last* time you had anal sex with a *man* was a condom used?	Yes<br>No	1<br>2	
**39**	If yes, why did you use a condom the last time you had sex with a man?	Avoid sexually transmitted infection/HIV<br>Partner's choice<br>Other (specify)____________________	1<br>2<br>3	
**40**	If no, why wasn't a condom used?<br>(circle more than one if required)	Not available<br>Too expensive<br>Partner objected<br>Don't know how to use one<br>Do not like using them<br>I trust my partner<br>I was drunk/using drugs<br>Condoms reduce pleasure<br>Condom's don't work<br>Goes against my religion<br>I don't care if I use one or not<br>Other (specify)____________________	1<br>2<br>3<br>4<br>5<br>6<br>7<br>8<br>9<br>10<br>11<br>12	
**41**	Is it possible for you to get a condom every time you need one?	Yes<br>No	1<br>2	
**42**	Where do you get condoms?<br>(circle more than one if required)	Shop<br>Pharmacy<br>Market/street vendor<br>Health facility (clinic/hospital/aid post)<br>VCT Centre<br>Condom dispenser<br>Friend<br>Family member<br>Fellow student<br>Fellow worker<br>At workplace<br>Other (specify)________________	1 →<br>2 →<br>3 →<br>4 →<br>5 →<br>6 →<br>7 →<br>8 →<br>9 →<br>10 →<br>11 →<br>12 →	**Once answered Skip Q 43 and Go to Q 44**
**43**	What is the main reason/s you have not had sex?<br>(circle more than one if required)	Not ready for sex<br>Afraid of pregnancy<br>Afraid of HIV/STIs<br>Afraid parents may find out<br>Afraid will be expelled from school/uni<br>Don't have enough confidence<br>Afraid of compensation<br>Too expensive<br>Religious beliefs<br>Waiting for the right person<br>Waiting until I get married<br>Not interested in sex<br>Other (Specify)________________	1<br>2<br>3<br>4<br>5<br>6<br>7<br>8<br>9<br>10<br>11<br>12<br>13	

**Section 4: Penile Modification**<br><br>In this section of the questionnaire you will be asked questions about different types of practices such as inserting or attaching objects to the penis.				
44	Have you ever inserted small objects or attached any objects to your penis?	Yes<br>No	1 →<br>2 →	**Go to 45**<br>**Go to 48**
45	What kinds of objects have you inserted or attached to your penis?	Wire<br>String<br>Ball bearings or other small objects<br>Plastic<br>Piece of toothbrush<br>Other (specify)___________________	1<br>2<br>3<br>4<br>5<br>6	
46	What was used to cut the skin when you put in the inserts?	Scalpel (long sharp blade)<br>Razor blade<br>Knife<br>Sharpened tooth brush<br>Sharpened bamboo<br>Sharp glass<br>Scissors<br>Other (specify)___________________	1<br>2<br>3<br>4<br>5<br>6<br>7<br>8	
47	Why did you insert or attach objects?<br>(circle more than one if required)	To make penis longer<br>To make penis wider<br>To make penis stronger<br>To make it harder<br>To have an insert<br>Create pleasure for men<br>Create pleasure for women<br>Punish women<br>Punish men<br>Punish self<br>Makes erection last longer<br>Delay ejaculation<br>Peer influence<br>Other (specify) ___________________	1<br>2<br>3<br>4<br>5<br>6<br>7<br>8<br>9<br>10<br>11<br>12<br>13<br>14	
48	Have you had any substance injected into your penis?	Yes<br>No	1 →<br>2 →	**Go to 49**<br>**Go to 52**
49	What substance was your penis injected with? Please explain<br>____<br>____			
50	Why was the injection done? Please explain<br>____<br>____			
51	What effect does the injection have? Please explain<br>____<br>____			
52	Have you done anything else to modify your penis eg washed with special soap, drink medicine<br>____<br>____<br>____			

Section 5: Knowledge of and Attitudes Towards Foreskin Cutting

In this section of the questionnaire you will be asked a series of questions about cutting or removing the foreskin.

The foreskin is the name of the skin that covers the end of the penis. In PNG a lot of men have had their foreskin cut. Some men have had their foreskin cut but not removed so the foreskin partially covers the head of the penis or hangs below the penis. In PNG this is commonly known as a ***split***. Some other men have had the entire foreskin removed so there is no foreskin at all at the end of the penis. In PNG this is commonly known as ***round cut***.
In this section of the questionnaire you will be asked your opinion of both split and round cut. We appreciate your honesty and assure you that none of this information will be linked to you as an individual.

Please Circle 1 (Yes) 2 (No) or 3 (Unsure) for each statement below

		Yes	No	Unsure
53	Having a split foreskin is a part of my custom/culture	1	2	3
54	Having a round cut is part of my custom/culture	1	2	3
55	The head of the penis should be covered with a foreskin	1	2	3
56	Having a split foreskin proves manhood	1	2	3
57	Having a round cut proves manhood	1	2	3
58	A split foreskin decreases sexual pleasure for a man	1	2	3
59	A round cut decreases sexual pleasure for a man	1	2	3
60	Men with a split foreskin are respected by their peers	1	2	3
61	Men with a round cut are respected by their peers	1	2	3
62	Sex lasts longer for men who have a split foreskin	1	2	3
63	Sex lasts longer for men who have a round cut	1	2	3
64	Pain from having the foreskin split or round cut is bearable for an adult male	1	2	3
65	Having the foreskin split or round cut in a village setting can result in the penis becoming infected by germs	1	2	3
66	Having the foreskin split or round cut in a village setting can result in a lot of blood loss	1	2	3
67	Having a split foreskin is forbidden by my religion	1	2	3
68	Having a round cut is forbidden by my religion	1	2	3
69	Having a split foreskin is forbidden by my custom/culture	1	2	3
70	Having a round cut is forbidden by my custom/culture	1	2	3
71	Having a split foreskin encourages men to have more sexual partners	1	2	3
72	Having a round cut encourages men to have more sexual partners	1	2	3
73	Men with a split foreskin do not need to use condoms to protect them from STI’s and HIV	1	2	3
74	Men with a round cut do not need to use condoms to protect them from STI’s and HIV	1	2	3
75	Having split foreskin is fashionable/stylish	1	2	3
76	Having a round cut is fashionable/stylish	1	2	3
77	Having a round cut in a clinic or hospital is expensive	1	2	3
78	It is safe to use the same blade or razor to split or remove the foreskin of many men at one time	1	2	3

		Yes	No	Unsure
78a	Splitting the foreskin in a village by a friend or relative is a safe procedure	1	2	3
79	Removing the foreskin in a village by a friend or relative is a safe procedure	1	2	3
80	Men with a split foreskin can become infected with HIV	1	2	3
81	Men with a round cut can become infected with HIV	1	2	3
82	My partner supports having a split foreskin	1	2	3
83	My partner supports having a round cut	1	2	3
84	My family support having a split foreskin	1	2	3
85	My family support having a round cut	1	2	3
86	Having a round cut by a doctor or nurse in a clinic or hospital is a safe procedure	1	2	3
87	A split foreskin reduces the risk of becoming infected with HIV	1	2	3
88	A round cut reduces the risk of becoming infected with HIV	1	2	3
89	Allowing blood to flow when the foreskin is split or removed is important in my custom/culture	1	2	3
90	Women prefer to have sex with a man who has a split foreskin	1	2	3
91	Women prefer to have sex with a man who has a round cut	1	2	3
92	A man needs to eat special food in the days after having his foreskin split or removed	1	2	3
93	A man needs to reduce the amount of water he drinks in the days after having his foreskin split or removed	1	2	3
94	A man needs to avoid women in the days after having his foreskin split or removed	1	2	3
95	A split foreskin helps keep the penis clean and healthy	1	2	3
96	A round cut helps keep the penis clean and healthy	1	2	3
97	A split foreskin makes a man's body grow strong	1	2	3
98	A round cut makes a man's body grow strong	1	2	3
99	A split foreskin makes the penis to grow bigger	1	2	3
100	A round cut makes the penis to grow bigger	1	2	3
101	It is easy to keep an uncircumcised penis clean	1	2	3
102	Pain from removing the foreskin is bearable for a child	1	2	3
103	Boys should have their foreskin removed soon after birth	1	2	3

Thank you for your answers to these questions!

There are three more sections in the questionnaire. However you only need to complete one of them. The section you need to go to next depends upon your answer to the next question.
Please complete the question on the next page and the follow the instructions.

104

Please circle the number beside the picture that looks most like your own foreskin and then follow instructions.

1. The foreskin has not been cut and completely covers the head of penis

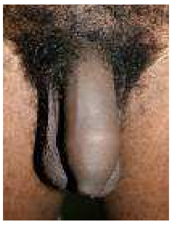

2. Foreskin has been cut but still partially covers the head of penis

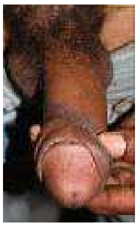

3. Foreskin has been cut and remains loose behind the head of the penis

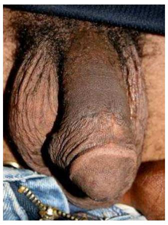

4. Foreskin has been cut on both sides leaving two or more tags

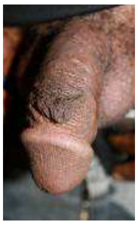

5. Foreskin has been cut with scarring along the penis

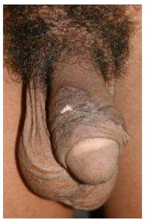

6. 'Cowboy cut' where foreskin can be pulled back over the head of the penis

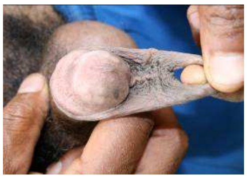

7. Total removal of the foreskin.

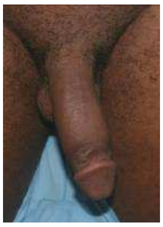

8. Other – please explain and/or draw picture (then go to relevant section)

____

____

If you circled Number 1: foreskin has never been cut ⟶ **Go to Section 6 (Page 10)**

If you circled Numbers 2, 3, 4, 5 or 6: any style of split ⟶ **Go to Section 7 (Page 12)**

If you circled Number 7: total removal of foreskin ⟶ **Go to Section 8 (Page 16)**

**Section 6: Questions for men who have not had any cuts to their foreskin.**<br><br>This section contains questions for men have never had their foreskin cut. We appreciate your honesty and assure you that none of this information will be linked to you as an individual.				
**105**	What is your main reason for not having had your foreskin split or removed? Please explain<br>__________<br>__________			
**106**	What do *men* in your community think about a man who his foreskin split or removed? Please explain<br>__________<br>__________			
**107**	What do *women* in your community think about a man who gets his foreskin split or removed? Please explain<br>__________<br>__________			
**108**	Would you have your foreskin removed if it had a health benefit?<br><br><br>Please explain<br>__________<br>__________	Yes<br>No<br>Maybe	1<br>2<br>3	
**109**	Would you have your foreskin removed if it reduced the risk of getting HIV?<br><br><br>Please explain your answer<br>__________<br>__________	Yes<br>No<br>Maybe	1<br>2<br>3	
**110**	If you decide to have your foreskin removed where would you go to have it done?<br>__________<br>__________			
**111**	If you decided to have your foreskin removed who would you want to do it?<br>__________<br>__________			
**112**	Are you planning to have your foreskin removed in the future?<br>Please explain<br><br>__________<br>__________	Yes<br>No<br>Maybe	1<br>2<br>3	
**113**	Do you recommend your friends have their foreskin removed?<br>Please explain:<br>__________<br>__________	Yes<br>No	1<br>2	

Questions 114 to 118 are for men who have children. If you do not have children please go to Question 119				
**114**	Have any of your male children had their foreskin split or removed?	Yes<br>No	1 →<br>2 →	**Go to 115**<br>**Go to 119**
**115**	Please explain how old were your male child was when he had his foreskin split or removed. If you have more than one male child please list the age of each child when his foreskin was cut.<br>__________<br>__________<br>__________<br>__________			
**116**	Why did your male child (or children) have their foreskin split or removed? Please explain:<br>__________<br>__________			
**117**	Where did your male child (or children) have their foreskin split or removed? (eg clinic, village) Please explain:<br>__________<br>__________			
**118**	Who performed the split or foreskin removal of your male child (or children) and what type of cut was done? Please explain:<br>__________<br>__________			
**119**	Would you have the foreskin removed from your male children if it had a health benefit?	Yes<br>No	1<br>2	
**120**	Would have the foreskin removed from your male children if it reduced the risk of HIV or STIs?	Yes<br>No	1<br>2	
**121**	Do you have anything else you would like to share with us about cutting or removing the foreskin?<br>__________<br>__________<br>__________<br>__________<br>__________<br>__________<br>__________			

The next stage of the study is for a health professional to examine your penis so that researchers can learn more about foreskin cutting. If you want to continue to this stage of the study please remove the sheet at the back page of this questionnaire and follow instructions about where and when to go for the clinical examination.

After removing the instruction sheet please place your completed questionnaire into the envelope provided and return to your Provincial Club Leader

Thank you for taking the time to complete this questionnaire

**Section 7: Questions for men who have any style of split foreskin**<br><br>This section contains questions for men who have had their foreskin cut but not removed so some of the foreskin remains. We appreciate your honesty and assure you that none of this information will be linked to you as an individual.				
**122**	What do *men* in your community think about a man who has his foreskin cut or removed?<br>____________________<br>____________________			
**123**	What do *women* in your community think about a man has his foreskin cut or removed?<br>____________________<br>____________________			
**124**	What are the main reasons you had your foreskin cut? (can be more than one)<br>____________________<br>____________________			
**125**	Describe the location where you have your foreskin cut? (eg in a clinic, in a village)<br>____________________<br>____________________			
**126**	Who performed the cut (e.g. doctor, nurse, friend, uncle)?<br>____________________<br>____________________			
**127**	Describe how was your foreskin cut (e.g. one cut along the top; one cut along each side)?<br>____________________<br>____________________			
**128**	Does your style of cut have a name?<br>____________________<br>____________________			
**129**	What tool or instrument was used to cut your foreskin?<br>____________________<br>____________________			
**130**	Was the tool used to cut your foreskin also used for someone else? (please explain)<br>____________________<br>____________________			
**131**	Was the cutting of your foreskin a part of an initiation?<br>Please explain<br>____________________<br>____________________	Yes<br>No	1<br>2	
**132**	Did the cutting of your foreskin have any religious or spiritual significance?<br>Please explain<br>____________________<br>____________________	Yes<br>No	1<br>2	

**133**	How old were you when your foreskin was cut?	I was ______________years old		
**134**	After your cut how did you know when your penis was fully healed?<br>______________________________<br>______________________________			
**135**	How many weeks did it take for the cut on your penis to heal?	It took _____________weeks		
**136**	Did you have sex with anyone before the cut was fully healed?	Yes<br>No	1<br>2	
**137**	If yes, did you use a condom?	Yes<br>No	1<br>2	
**138**	Were there any special things you needed to do to prepare to have your foreskin cut?<br>Please Explain<br>______________________________<br>______________________________	Yes<br>No	1<br>2	
**139**	Were there any special things you needed to do immediately after having your foreskin cut?<br>Please Explain<br>______________________________<br>______________________________	Yes<br>No	1<br>2	
**140**	When you had your foreskin cut did you have any complications? (eg bleeding, pain)<br>Please explain<br>______________________________<br>______________________________	Yes<br>No	1<br>2	
**141**	Do you have any regrets about having your foreskin cut?<br>Please explain<br>______________________________<br>______________________________	Yes<br>No	1<br>2	
**142**	Does having your foreskin cut help you feel more like a man?<br>Please explain<br>______________________________<br>______________________________	Yes<br>No	1<br>2	
**143**	Has your sex life changed since you had your foreskin cut?<br>Please explain<br>______________________________<br>______________________________	Yes<br>No	1<br>2	
**144**	Did having your foreskin cut change the number of sexual partners you have had?<br>Please explain<br>______________________________<br>______________________________	Yes<br>No	1<br>2	

**145**	After having your foreskin cut have you found sex:<br><br>More enjoyable than before<br>Less enjoyable than before<br>About the same as before<br>Don't know as had not had sex before cut<br>Don't know as have not had sex after cut<br>Don't know as have never had sex	 <br>1<br>2<br>3<br>4<br>5<br>6	
Questions 146 to 150 are for men who have children. If you do not have children please go to Question 151			
**146**	Have any of your male children had their foreskin split or removed? — Yes<br>No	1 →<br>2 →	**Go to 147**<br>**Go to 151**
**147**	Please explain how old were your male child was when he had his foreskin cut or removed. If you have more than one male child please list the age of each child when his foreskin was cut.<br>____________________<br>____________________<br>____________________<br>____________________		
**148**	Why did your male child (or children) have their foreskin cut or removed? Please explain:<br>____________________<br>____________________		
**149**	Where did your male child (or children) have their foreskin cut or removed? (eg clinic, village) Please explain:<br>____________________<br>____________________		
**150**	Who performed the cut or foreskin removal of your male child (or children) and what type of cut was done? Please explain:<br>____________________<br>____________________		
**151**	Would you have the foreskin removed from your male children if it had a health benefit? — Yes<br>No	1<br>2	
**152**	Would have the foreskin removed from your male children if it reduced the risk of HIV or STIs? — Yes<br>No	1<br>2	
**153**	Would you have your foreskin totally removed if it had a health benefit? Yes<br>Please explain No<br>____________________<br>____________________<br>____________________	1<br>2	
**154**	Would you have your foreskin totally removed if it would reduce the risk of getting HIV?<br>Please explain Yes<br>No<br>____________________<br>____________________<br>____________________	 1<br>2	

**155**	If you decided to have your foreskin totally removed where would you go to have this done?<br>______________________________<br>______________________________		
**156**	If you decided to have your foreskin totally removed who would you want to do it?<br>______________________________<br>______________________________		
**157**	Are you planning to have your foreskin totally removed in the future? Yes<br>Please explain No<br>Maybe<br>______________________________<br>______________________________	1<br>2<br>3	
**158**	Do you recommend the total removal of the foreskin to your friends? Yes<br>Please explain No<br>______________________________<br>______________________________	1<br>2	
**159**	Do you have anything else you would like to share with us about male circumcision or foreskin cutting?<br>______________________________		
	The next stage of the study is for a health professional to examine your penis so that researchers can learn more about foreskin cutting. If you want to continue to this stage of the study please remove the sheet at the back page of this questionnaire and follow instructions about where and when to go for the clinical examination.<br><br>**After removing the instruction sheet please place your completed questionnaire into the envelope provided and return to your provincial club leader**<br><br>**Thank you for taking the time to complete this questionnaire**		

Section 8 Questions for men who have had their foreskin fully removed.<br><br>This section contains questions for men who have had their foreskin fully removed.<br>We appreciate your honesty and assure you that none of this information will be linked to you as an individual.			
**160**	What do *men* in your community think about a man who has his foreskin cut or removed?<br>____________________<br>____________________		
**161**	What do *women* in your community think about a man who has his foreskin cut or removed?<br>____________________<br>____________________		
**162**	What are the main reasons why you had your foreskin removed? (can be more than)<br>____________________<br>____________________		
**163**	Describe the location where you have your foreskin removed? (eg in a clinic, in a village)<br>____________________<br>____________________		
**164**	Who removed your foreskin (e.g. doctor, nurse, friend, uncle)?<br>____________________<br>____________________		
**165**	Does your style of cut have a name?<br>____________________<br>____________________		
**166**	What tool or instrument was used to cut your foreskin?<br>____________________<br>____________________		
**167**	Was the tool used to cut your foreskin also used for someone else? (please explain)<br>____________________<br>____________________		
**168**	Was the removal of your foreskin a part of an initiation? Yes<br>Please explain No<br>____________________<br>____________________	1<br>2	
**169**	Did the removal of your foreskin have any religious or spiritual significance? Yes<br>Please explain No<br>____________________<br>____________________	1<br>2	
**170**	How old were you when your foreskin was removed? — I was _____________years old		

171	After your foreskin was removed how did you know when your penis was fully healed?<br>____<br>____		
172	How many weeks did it take for the cut on your penis to heal? It took ____________weeks		
173	Did you have sex with anyone before the cut was fully healed? Yes<br>No	1<br>2	
174	If yes, did you use a condom? Yes<br>No	1<br>2	
175	Were there any special things you needed to do to prepare to have your foreskin removed? Yes<br>Please Explain No<br>____<br>____	1<br>2	
176	Were there any special things you needed to do immediately after having your foreskin removed? Yes<br>Please Explain No<br>____<br>____	1<br>2	
177	When you were circumcised did you have any complications? (eg bleeding, pain) Yes<br>Please explain No<br>____<br>____	1<br>2	
178	Do you have any regrets about having your foreskin removed? Yes<br>Please explain No<br>____<br>____	1<br>2	
179	Does having your foreskin removed help you feel more like a man? Yes<br>Please explain No<br>____<br>____	1<br>2	
180	Has your sex life changed since having your foreskin removed? Yes<br>Please explain No<br>____<br>____	1<br>2	
181	Did having your foreskin removed change the number of number of sexual partners you have had? Yes<br>Please explain No<br>____<br>____<br>____	1<br>2	
182	After having your foreskin removed have you found sex:<br>More enjoyable than before<br>Less enjoyable than before<br>About the same as before<br>Don't know as had not had sex before having foreskin removed<br>Don't know as have not had sex after having foreskin removed<br>Don't know as have never had sex	 1<br>2<br>3<br>4<br>5<br>6	

Questions 183 to 187 are for men who have children. If you do not have children please go to Question 188				
**183**	Have any of your male children had their foreskin split or removed?	Yes<br>No	1 →<br>2 →	**Go to 184**<br>**Go to 185**
**184**	Please explain how old was your male child was when he had his foreskin cut or removed. If you have more than one male child please list the age of each child when his foreskin was cut.<br>__________<br>__________<br>__________<br>__________			
**185**	Why did your male child (or children) have their foreskin cut or removed? Please explain:<br>__________<br>__________			
**186**	Where did your male child (or children) have their foreskin cut or removed? (eg clinic, village) Please explain:<br>__________<br>__________			
**187**	Who performed the cut or foreskin removal of your male child (or children) and what type of cut was done? Please explain:<br>__________<br>__________			
**188**	Would you have the foreskin removed from your male children if it had a health benefit?	Yes<br>No	1<br>2	
**189**	Would have the foreskin removed from your male children if it reduced the risk of HIV or STIs?	Yes<br>No	1<br>2	
**190**	Do you recommend the full removal of the foreskin to your friends?<br>Please explain<br>__________<br>__________	Yes<br>No	1<br>2	
**191**	Do you have anything else you would like to share with us about male circumcision or foreskin cutting?<br>__________<br>__________<br>__________<br>__________			
	The next stage of the study is for a health professional to examine your penis so that researchers can learn more about foreskin cutting. If you want to continue to this stage of the study please remove the sheet at the back page of this questionnaire and follow instructions about where and when to go for the clinical examination.<br><br>**After removing the instruction sheet please place your completed questionnaire into the envelope provided and return to your Provincial Club Leader**<br><br>**Thank you for taking the time to complete this questionnaire**			

Instruction Sheet for Clinical Examination

Thank you for completing the 'Male Circumcision for HIV Prevention in PNG Study' questionnaire

To continue to the final part of the study please read the following instructions.

If you wish to volunteer to continue in the study, the next stage is for a health professional to examine your genital area. This is for all men regardless if you have never had your foreskin cut, had any style of split or had your foreskin totally removed. A medical examination is a part of the study so the researchers can learn more about foreskin cutting. The health professional may request to take a photograph of your genital area. These photographs will be identified by numbers only – no names will be recorded. Please tear off this page from the questionnaire and take it with you. You will notice there is a participant number below. This is to make sure the information from the clinical examination is linked to the information in the questionnaire. Your name will not be recorded and no information will be able to be linked with you as an individual.

Participant Number ☐

Clinical examination will take place at 2 locations

1. XXX
2. XXX
3.
    Day 1.   XX
    Day: 2. XX
    Day: 3. XX

Please take this instruction sheet with you to the clinic and give it to the health professional at either of the above locations on the dates and times specified (above)

You may ask questions or seek medical assistance from the health professional if you have any other health issues or problems.

If you have any questions please contact

Ms Rachael Tommbe
Pacific Adventist University
Phone:+675 328 0342
Email: Rachael.Tommbe@pau.ac.pg

Dr Clement Manineng
Divine Word University
Phone: +675 424 1887
Email: cmanineng@dwu.ac.pg

Dr David MacLaren
James Cook University
Phone: +61 (7) 4042 1658
Email: david.maclaren@jcu.edu.au
