# Supplementary material for: Foreskin cutting beliefs and practices and the acceptability of male circumcision for HIV prevention in Papua New Guinea
Source: BMC Public Health. 2013 Sep 9;13:818. doi: 10.1186/1471-2458-13-818 (PMC3846639; doi:10.1186/1471-2458-13-818)
Supplement: Additional file 2: Figure S2 — Female Questionnaire in English. [file 1471-2458-13-818-S2.pdf]

Acceptability of Male Circumcision for HIV Prevention in PNG Study

JAMES COOK UNIVERSITY AUSTRALIA — PAU Pacific Adventist University EDUCATE TO SERVE — Divine Word University Madang

A Joint JCU, PAU, DWU Study 2010-2012: NHMRC Project Grant 601003

Questionnaire for Female Participants

Participant Number [ ]

You are invited to take part in a study that is seeking to find out if increasing the number of men who are circumcised would be an acceptable way to reduce the spread of HIV in Papua New Guinea (PNG). This is important because it has been shown in some other countries that men who are circumcised are less likely to contract HIV than men who are not circumcised. The study will ask both men and women 18 years and over about their experience and attitudes about male circumcision. Your participation in the study as a woman will help us understand the possible acceptability of male circumcision for HIV prevention in PNG. The study is being conducted by **Dr David MacLaren** from **James Cook University**, in partnership with researchers from **Pacific Adventist University** and **Divine Word University**

You are invited to complete this self-administered questionnaire. Questions in it are specifically for women and ask about your thoughts, as a woman, about male circumcision. By completing and returning this questionnaire you are giving consent for the results to be used in the study. The questionnaire will take you between 30-45 minutes to complete. Your name will not be recorded on the questionnaire and your responses will not be able to be linked to you as an individual.

Do I have to take part of this study?
Taking part in this study is completely voluntary and you can stop taking part in the study at any stage without explanation or prejudice. You may also withdraw any data from the study before it is analysed.

This is a very personal subject: What if I become distressed?
As this study is about sexual health and is of a sensitive nature, some people may find some of the questions a little distressing. If you do feel upset or distressed in any way, please advise the researcher or the contacts at the bottom of this sheet and you will be referred to someone who can help you.

What will happen to the results of the study?
Your responses and identity will be kept strictly confidential. The data from the study will be used in research publications and reports to PNG Ministry of Heath, PNG National AIDS Council and Provincial AIDS Committees, other health researchers and health service providers. You will not be identified in any way in these publications.

**Dr David MacLaren**	**Ms Rachael Tommbe**	**Dr Clement Manineng**
**James Cook University**	**Pacific Adventist University**	**Divine Word University**
**Phone: +61 (7) 4042 1658**	**Phone:+675 328 0342**	**Phone: +675 424 1887**
**Email:** david.maclaren@jcu.edu.au	**Email:** Rachael.Tommbe@pau.ac.pg	**Email:** cmanineng@dwu.ac.pg

Section 1: Background Characteristics

In this first section of the questionnaire you will be asked some questions about your background.
Please circle the number beside your answer or complete the statement beside the question.
After some questions there are instructions to tell you which question to go to next. You will see instructions in the 'Go To' column. If there are no instructions in the 'Go To' column please continue to the next question.

No.	Questions			Go To
1	What is your gender	Male<br>Female	1<br>2	
2	How old are you?	I am ____________years old		
3	Which Province do you come from?<br>(circle more than one if required)	Bougainville (ARB)<br>Central<br>East New Britain<br>East Sepik<br>Eastern Highlands<br>Enga<br>Gulf<br>Hela<br>Jiwaka<br>Madang<br>Manus<br>Milne Bay<br>Morobe<br>NCD<br>New Ireland<br>Oro<br>Sandaun<br>Simbu<br>Southern Highlands<br>West New Britain<br>Western<br>Western Highlands<br>Other(specify)________________	1<br>2<br>3<br>4<br>5<br>6<br>7<br>8<br>9<br>10<br>11<br>12<br>13<br>14<br>15<br>16<br>17<br>18<br>19<br>20<br>21<br>22<br>23	
4	What is your religion?	Christian<br>Hindu<br>Muslim<br>None<br>Other ______________________	1 →<br>2 →<br>3 →<br>4 →<br>5 →	**Go to Q 5**<br>**Go to Q 6**<br>**Go to Q 6**<br>**Go to Q 6**<br>**Go to Q 6**
5	What Christian denomination do you belong to?	Anglican<br>AOG<br>Baptist<br>Catholic<br>Christian Brethren Church<br>ECB<br>Evangelical Churches<br>Jehovah's Witness<br>Lutheran<br>Mormon<br>Pentecostal<br>PNG Bible Church<br>Revival<br>SDA<br>United<br>Other(specify)________________<br>None	1<br>2<br>3<br>4<br>5<br>6<br>7<br>8<br>9<br>10<br>11<br>12<br>13<br>14<br>15<br>16<br>17	
6	What is your current marital status?	Single<br>Married<br>Separated<br>Divorced<br>Widow<br>Other (specify)________________	1<br>2<br>3<br>4<br>5<br>6	

**7**	How many wives does your husband have? (if not married mark ‘0’)	My husband has__________wives		
**8**	How many male children do you have? (if no male children mark ‘0’)	I have ____________male children		
**9**	How many female children do you have? (in no female children mark ‘0’)	I have __________female children		
**10**	What is the highest level of formal education you have completed?	Have not attended school<br>Elementary (prep – elementary 2)<br>Primary (grade 3-8)<br>High School (grade 9-10)<br>Secondary School (grade 11-12)<br>Vocational<br>Technical/College<br>University<br>Other<br>(specify)__________________	1<br>2<br>3<br>4<br>5<br>6<br>7<br>8<br>9	
**11**	Are you currently enrolled in a school, college or university?	Yes<br>No	1 →<br>2 →	**Go to Q12**<br>**Go to Q13**
**12**	What year level are you currently studying?	High School (grade 9-10)<br>Secondary School (grade 11-12)<br>Vocational/College Year 1<br>Vocational/College Year 2<br>Vocational/College Year 3<br>University Year 1<br>University Year 2<br>University Year 3<br>University Year 4<br>University Postgraduate<br>Other (specify)_______________	1<br>2<br>3<br>4<br>5<br>6<br>7<br>8<br>9<br>10<br>11	
**13**	What do you do to earn money?	Self employed e.g. grow crops<br>Manual work/field worker<br>Trade or technical work<br>Professional work<br>Dependant on family/guardian<br>Student Scholarship<br>Other (specify)________________	1<br>2<br>3<br>4<br>5<br>6<br>7	

Section 2: Knowledge of HIV

In this section of the questionnaire you will be asked a series of questions about HIV and how it is transmitted

**14**	How much do you know about HIV?	Nothing at all<br>A little<br>Moderate amount<br>A lot	1<br>2<br>3<br>4	
**15**	Where does your information about HIV come from? (circle more than one if required)	Radio<br>TV<br>News paper<br>Non Government Organisation<br>Religious Organisation<br>Teacher<br>Friend<br>Relative<br>Doctor/Nurse/Health Worker<br>VCT Centre<br>Clinic/Health Centre<br>Pamphlet/brochure<br>Poster<br>Internet<br>Others (specify) _____________	1<br>2<br>3<br>4<br>5<br>6<br>7<br>8<br>9<br>10<br>11<br>12<br>13<br>14<br>15	

**16**	Can a woman get HIV from having sex with a man who has HIV?	Yes<br>No<br>Not sure	1<br>2<br>3	
**17**	Can a man get HIV from having sex with a woman who has HIV?	Yes<br>No<br>Not sure	1<br>2<br>3	
**18**	Can a man get HIV from having sex with a man who has HIV?	Yes<br>No<br>Not sure	1<br>2<br>3	
**19**	Can a person get HIV from a needle, razor or other cutting tools that have already been used by someone else for tattooing, scarification or circumcision?	Yes<br>No<br>Not sure	1<br>2<br>3	
**20**	Can a person get HIV from mosquito bites?	Yes<br>No<br>Not sure	1<br>2<br>3	
**21**	If someone with HIV coughs or sneezes near another person, can that person get HIV?	Yes<br>No<br>Not sure	1<br>2<br>3	
**22**	Can a person get HIV by hugging someone who has HIV?	Yes<br>No<br>Not sure	1<br>2<br>3	
**23**	Can a person get HIV by sharing a meal with someone who has HIV?	Yes<br>No<br>Not sure	1<br>2<br>3	
**24**	If a woman with HIV is pregnant, can her baby become infected with HIV?	Yes<br>No<br>Not sure	1<br>2<br>3	
**25**	Can the family planning pill protect a woman from HIV infection?	Yes<br>No<br>Not sure	1<br>2<br>3	
**26**	If condoms are used correctly during sex do they help protect people from getting HIV?	Yes<br>No<br>Not sure	1<br>2<br>3	
**27**	Can someone who looks healthy have HIV?	Yes<br>No<br>Not sure	1<br>2<br>3	

Section 3: History of Sexual Practices
In this section of the questionnaire you will be asked some very personal questions about sex. We appreciate your honesty and assure you that none of this information will be linked to you as an individual.

**28**	Have you ever had sex with a man at any time in your life? By that we mean vaginal or anal penetrative sex.	Yes<br>No	1 →<br>2 →	**Go to Q29**<br>**Go to Q38**
**29**	How old were you when you first had sex?	I was ____________years old		
**30**	The *first* time you had sex, how old was the man you had sex with?<br>(If unsure of exact age please estimate)	The person was __________years old		
**31**	Have you ever used a condom with a sexual partner at any time in your life?	Yes<br>No	1<br>2	
**32**	How many men have you had sex with in your life? (If unsure of exact number of men please estimate)	I have had sex with ___________men in my life		
**33**	The *last* time you had sex with a man was a condom used?	Yes<br>No	1<br>2	
**34**	If Yes, why did you use a condom the last time you had sex with a man?	Avoid pregnancy<br>Avoid sexually transmitted infection/HIV<br>Partner's choice<br>Other (specify)______________________	1<br>2<br>3<br>4	

**35**	If No, why wasn't a condom used?<br>(circle more than one if required)	Not available<br>Too expensive<br>Partner objected<br>Don't know how to use one<br>Do not like using them<br>I trust my partner<br>I was drunk/using drugs<br>Condoms reduce pleasure<br>Condom's don't work<br>Goes against my religion<br>I don't care if I use one or not<br>Other (specify)_____________________	1<br>2<br>3<br>4<br>5<br>6<br>7<br>8<br>9<br>10<br>11<br>12	
**36**	Is it possible for you to get a condom every time you need one?	Yes<br>No	1<br>2	
**37**	Where do you get condoms?<br>(circle more than one if required)	Shop<br>Pharmacy<br>Market/street vendor<br>Health facility (clinic/hospital/aid post)<br>VCT Centre<br>Condom dispenser<br>Friend<br>Family member<br>Fellow student<br>Fellow worker<br>At workplace<br>Other (specify)__________________	1 →<br>2 →<br>3 →<br>4 →<br>5 →<br>6 →<br>7 →<br>8 →<br>9 →<br>10 →<br>11 →<br>12 →	**Once answered Skip Q 38 and Go to Q 39**
**38**	What is the main reason/s you have not had sex?<br>(circle more than one if required)	Not ready for sex<br>Afraid of pregnancy<br>Afraid of HIV/STIs<br>Afraid parents may find out<br>Afraid will be expelled from school/uni<br>Don't have enough confidence<br>Afraid of compensation<br>Too expensive<br>Religious beliefs<br>Waiting for the right person<br>Waiting until I get married<br>Not interested in sex<br>Other (Specify)_________________	1<br>2<br>3<br>4<br>5<br>6<br>7<br>8<br>9<br>10<br>11<br>12<br>13	

Section 4: Knowledge of and Attitudes Towards Foreskin Cutting

The foreskin is the name of the skin that covers the end of the penis. In PNG a lot of men have had their foreskin cut.
Some men have had their foreskin cut but not removed so the foreskin partially covers the head of the penis or hangs below the penis. In PNG this is commonly known as a ***split***. Some other men have had the entire foreskin removed so there is no foreskin at all at the end of the penis. In PNG this is commonly known as a ***round cut***.
In this section of the questionnaire you will be asked your opinion of both a split and a round cut. We appreciate your honesty and assure you that none of this information will be linked to you as an individual.

Please Circle 1 (Yes) 2 (No) or 3 (Unsure) for each statement below

		Yes	No	Unsure
**39**	Having a split foreskin is a part of my custom/culture	**1**	**2**	**3**
**40**	Having a round cut is part of my custom/culture	**1**	**2**	**3**
**41**	The head of the penis should be covered with a foreskin	**1**	**2**	**3**
**42**	Having a split foreskin proves manhood	**1**	**2**	**3**
**43**	Having a round cut proves manhood	**1**	**2**	**3**
**44**	A split foreskin decreases sexual pleasure for a man	**1**	**2**	**3**
**45**	A round cut decreases sexual pleasure for a man	**1**	**2**	**3**
**46**	Men with a split foreskin are respected by their peers	**1**	**2**	**3**

		Yes	No	Unsure
47	Men with a round cut are respected by their peers	1	2	3
48	Sex lasts longer for men who have a split foreskin	1	2	3
49	Sex lasts longer for men who have a round cut	1	2	3
50	Pain from having the foreskin split or round cut is bearable for an adult male	1	2	3
51	Having the foreskin split or round cut in a village setting can result in the penis becoming infected by germs	1	2	3
52	Having the foreskin split or round cut in a village setting can result in a lot of blood loss	1	2	3
53	Having the foreskin split is forbidden by my religion	1	2	3
54	Having a round cut is forbidden by my religion	1	2	3
55	Having the foreskin split is forbidden by my custom/culture	1	2	3
56	Having a round cut is forbidden by my custom/culture	1	2	3
57	Having a split foreskin encourages men to have more sexual partners	1	2	3
58	Having a round cut encourages men to have more sexual partners	1	2	3
59	Men with a split foreskin do not need to use condoms to protect them from STI's and HIV	1	2	3
60	Men with a round cut do not need to use condoms to protect them from STI's and HIV	1	2	3
61	Having split foreskin is fashionable/stylish	1	2	3
62	Having a round cut is fashionable/stylish	1	2	3
63	Having a round cut in a clinic or hospital is expensive	1	2	3
64	It is safe to use the same blade or razor to split or remove the foreskin of many men at one time	1	2	3
65	Splitting the foreskin in a village by a friend or relative is a safe procedure	1	2	3
66	Removing the foreskin in a village by a friend or relative is a safe procedure	1	2	3
67	Men with a split foreskin can become infected with HIV	1	2	3
68	Men with a round cut can become infected with HIV	1	2	3
69	My partner supports having a split foreskin	1	2	3
70	My partner supports having a round cut	1	2	3
71	My family support having a split foreskin	1	2	3
72	My family support having a round cut	1	2	3
73	Having a round cut by a doctor or nurse in a clinic or hospital is a safe procedure	1	2	3
74	A split foreskin reduces the risk of becoming infected with HIV	1	2	3
75	A round cut reduces the risk of becoming infected with HIV	1	2	3
76	Allowing blood to flow when the foreskin is slit or removed is important in my custom/culture	1	2	3
77	Women prefer to have sex with a man who has a split foreskin	1	2	3
78	Women prefer to have sex with a man who has a round cut	1	2	3
79	A man needs to eat special food in the days after having his foreskin split or removed	1	2	3
80	A man needs to reduce the amount of water he drinks in the days after having his foreskin split or removed	1	2	3
81	A man needs to avoid women in the days after having his foreskin split or removed	1	2	3
82	A split foreskin helps keep the penis clean and healthy	1	2	3
83	A round cut helps keep the penis clean and healthy	1	2	3
84	A split foreskin makes a man's body grow strong	1	2	3
85	A round cut makes a man's body grow strong	1	2	3
86	A split foreskin makes the penis to grow bigger	1	2	3
87	A round cut makes the penis to grow bigger	1	2	3
88	It is easy to keep an uncircumcised penis clean	1	2	3
89	Pain from removing the foreskin is bearable for a child	1	2	3
90	Boys should have their foreskin removed soon after birth	1	2	3

**Section 5: Further Questions for women about male circumcision**<br>In this section of the questionnaire there are questions for you as a woman to help us understand your attitudes towards male circumcision. These questions ask about your ideas of circumcision of men as sexual partners and male children. We appreciate your honesty and assure you that none of this information will be linked to you as an individual.	
**91**	What do *women* in your community think about a man who has a split or a round cut? Please explain<br>__________<br>__________<br>__________<br>__________
**92**	What do *men* in your community think about a man who has a split or a round cut? Please explain<br>__________<br>__________<br>__________<br>__________
**93**	Do women prefer men as sexual partners who have a split or a round cut? Please explain<br>__________<br>__________<br>__________<br>__________
**94**	In your opinion, what would be the positive changes for a woman if her sexual partner had a round cut? Please explain<br>__________<br>__________<br>__________<br>__________
**95**	In your opinion, what would be the negative changes for a woman if her sexual partner had a round cut? Please explain<br>__________<br>__________<br>__________<br>__________<br>__________

**Questions 96 to 100** are for women who have children. If you do not have children please **go to Question 101**				
**96**	Have any of your male children had their foreskin split or removed?	Yes<br>No	1 →<br>2 →	**Go to Q94**<br>**Go to Q101**
**97**	Please explain how old were your male child was when he had his foreskin split or removed. If you have more than one male child please list the age of each child when his foreskin was cut.<br>____________________<br>____________________<br>____________________<br>____________________			
**98**	Why did your male child (or children) have their foreskin split or removed? Please explain:<br>____________________<br>____________________			
**99**	Where did your male child (or children) have their foreskin split or removed? (eg clinic, village) Please explain:<br>____________________<br>____________________			
**100**	Who performed the split or round cut on your male child (or children)? Please explain:<br>____________________<br>____________________			
**101**	Would you have your male child/children circumcised if it had a health benefit?	Yes<br>No<br>Unsure	1<br>2<br>3	
**102**	Would you have your male child/children circumcised if it reduced the risk of HIV or STIs?	Yes<br>No<br>Unsure	1<br>2<br>3	
**103**	Do you have anything else you would like to share with us about male circumcision or foreskin cutting?<br>____________________<br>____________________<br>____________________<br>____________________<br>____________________<br>____________________<br>____________________<br>____________________<br>____________________<br>____________________<br>____________________<br>____________________<br>Thank you for taking the time to complete this survey<br>**Please place it in the sealed envelope and return to your** *provincial club leader*			
